# Combination of Low-Dose Sulforaphane and Docetaxel on Mitochondrial Function and Metabolic Reprogramming in Prostate Cancer Cell Lines

**DOI:** 10.3390/ijms26031013

**Published:** 2025-01-24

**Authors:** Ana Peñata-Taborda, Pedro Espitia-Pérez, Lyda Espitia-Pérez, Andrés Coneo-Pretelt, Hugo Brango, Dina Ricardo-Caldera, Gean Arteaga-Arroyo, Luisa Jiménez-Vidal, Claudia Galeano-Páez, Karina Pastor-Sierra, Alicia Humanez-Alvarez, Osnamir Bru-Cordero, Nathalia Jones-Cifuentes, Bladimiro Rincón-Orozco, Stelia Mendez-Sanchez, Mario Negrette-Guzmán

**Affiliations:** 1 Grupo de Investigación Biomédicas y Biología Molecular, Universidad del Sinú E.B.Z., Montería 230001, Colombia; investigador01gibm@unisinu.edu.co (A.P.-T.); pedrojespitia@unisinu.edu.co (P.E.-P.); aconeo79@gmail.com (A.C.-P.); geanarteaga@unisinu.edu.co (G.A.-A.); lufejivi@gmail.com (L.J.-V.); claudiagaleano@unisinu.edu.co (C.G.-P.); karinapastor@unisinu.edu.co (K.P.-S.); aliciahumanez@unisinu.edu.co (A.H.-A.); 2 Facultad de Educación y Ciencias, Departamento de Matemáticas, Universidad de Sucre, Sincelejo 700003, Colombia; hugo.brango@unisucre.edu.co; 3 Grupo de Investigación Enfermedades Tropicales y Resistencia Bacteriana, Universidad del Sinú E.B.Z., Montería 230001, Colombia; dinaricardoc@unisinu.edu.co; 4 Dirección Académica, Universidad Nacional de Colombia, Kilómetro 9, Vía Valledupar-La Paz, La Paz 202010, Colombia; oebruc@unal.edu.co; 5 Departamento de Ciencias Básicas, Escuela de Medicina, Universidad Industrial de Santander, Bucaramanga 680002, Colombia; nathalia.jones.cifuentes@gmail.com (N.J.-C.); blrincon@uis.edu.co (B.R.-O.); maneguz@uis.edu.co (M.N.-G.); 6 Escuela de Química, Universidad Industrial de Santander, Bucaramanga 680002, Colombia; scmendez@uis.edu.co

**Keywords:** docetaxel, sulforaphane, prostate cancer, combination therapy, glycolysis, redox status, mitochondrial function

## Abstract

Considering the limitations of monotherapies due to chemoresistance and side effects, this research aimed to determine whether low doses of sulforaphane (SFN) combined with docetaxel (DCT) could enhance therapeutic efficacy. Prostate cancer cell lines LNCaP and PC-3 were treated with individual IC50 doses of SFN and DCT and half-reduced IC50 values for the SFN:DCT combination. Metabolic markers, including glucose consumption, lactate production, reactive oxygen species (ROS), mitochondrial mass, and caspase activity, were assessed. In LNCaP cells, the SFN:DCT combination reduced cell viability to 50%, comparable to DCT monotherapy (48%). Caspase 3 activation was also higher with SFN:DCT (2.4 ± 0.75 RFU) than DCT alone (2.1 ± 0.47 RFU), while caspase 8 activation remained comparable, indicating equivalent effectiveness at lower concentrations. In PC-3 cells, the combination induced caspase 3 activation (1.16 ± 0.0484 RFU) at levels slightly lower than DCT (1.51 ± 0.2062 RFU) but achieved greater reductions in mitochondrial mass, reflecting its ability to target metabolic vulnerabilities in aggressive phenotypes. Our findings suggest that the SFN:DCT combination is a promising strategy for early-stage prostate cancer. By achieving comparable efficacy to DCT monotherapy at low doses, the SFN:DCT combination maintains the therapeutic impact, mitigating the adverse effects of conventional DCT treatment.

## 1. Introduction

Prostate cancer is the fifth leading cause of death worldwide, being the second most common type of cancer diagnosed in men, according to the International Agency for Research on Cancer (IARC) [1]. Furthermore, according to estimations of the WHO Global Cancer Observatory, an increase in cases of prostate cancer is expected by 2040, particularly in undeveloped countries in Africa, the Americas, and the Caribbean [2]. The primary treatment for metastatic prostate cancer is androgen deprivation therapy (ADT) [3]. Although over 90% of patients respond to this treatment, the disease often progresses. Cancer adapts to a low testosterone environment, leading to a clinical state called castration-resistant prostate cancer (CRPC), characterized by an androgen-independent phenotype with a limited response to ADT, visceral and bone metastases, and relatively low levels of prostate-specific antigen (PSA) [4]. The standard chemotherapy scheme for patients with advanced prostate cancer and CRPC involves using taxanes such as cabazitaxel and docetaxel (DCT) [5]. Taxane molecular mechanisms include microtubule binding and stabilization, which inhibit proliferation. Additionally, taxanes prevent nuclear translocation of the androgen receptor by binding microtubule and dynein microtubule-associated motor protein [5].

However, over the last few years, in cases of CRPC, the taxane approach has achieved only a discrete survival improvement and an amelioration of analgesic control in the final phase of prostate cancer [6,7]. Particularly, DCT application produces adverse acute and chronic effects in the patient, generating myelosuppression [8], bone marrow dysfunction, anemia, thrombocytopenia, febrile neutropenia, and other unfavorable physiological manifestations, affecting the quality of life of patients with DCT and ADT in the long term [9].

The chemoresistance to conventional antineoplastic drugs is usually mediated by pathways regulated by apoptosis, cell adhesion, energy metabolism, and an adaptive response to oxidative stress [10]. An “altered redox” state is a hallmark of most tumors, including prostate cancer, characterized by increased ROS levels, enhanced antioxidant responses, and a dynamic shift between redox states during progression [11,12]. This ability to adapt redox states, present both in the early stages and throughout tumor progression, contributes to increased aggressiveness [11,12,13]. In certain types of cancer, this aggressiveness is accompanied by a heightened dependence on glycolysis, even under aerobic conditions, a phenomenon known as the Warburg effect [11,12,13]. Furthermore, oxidative stress induced by ROS production affects the onset, progression, and chemotherapeutic effects of prostate cancer treatment [14]. Thus, biologically active molecules capable of modulating the redox and glycolytic pathways in prostate cancer constitute important alternative therapeutics, sensitizing tumors and protecting unaffected tissues, and finally improving the therapeutic capacity of conventional antineoplastic drugs [10].

Sulforaphane (SFN) is a compound derived from cruciferous vegetables that induces an antioxidant cellular response associated with antitumor effects against different types of cancer, including cancers of the prostate, pancreas, breast, lung, ovarian, bladder, and colorectal cancers [15,16,17,18,19,20,21,22]. SFN exerts its therapeutic action through a variety of mechanisms, including detoxification of carcinogens and oxidants through modulation of phase I and II metabolic enzymes and cell cycle arrest in the G2/M and G1 phases to inhibit cell proliferation by inducing apoptosis. More recently evidenced is SFN-induced activation of the transcription factor Nrf2 (nuclear factor erythroid-2), a master regulator of cellular resistance to oxidative stress, through the expression of genes encoding the phase II detoxifying enzymes such as SOD, NQO1, HO-1, and HMOX1 [23].

Previous research showed that SFN could decrease the growth of human prostate cells, including non-tumorigenic BPH-1 epithelial cells and prostate cancer cells LNCaP and PC-3, by inhibiting histone deacetylase activity [24], as well as inducing caspase-mediated apoptosis, inhibiting the growth of PC-3 xenografts in nude mice [25]. On the other hand, even when Nrf2 plays a crucial role in the resistance of cancer cells to chemotherapy [26], it also modulates genes associated with antioxidant response elements (ARE). The binding of Nrf2 to ARE sequences activates a mitochondrial biogenesis program [27,28]. Through induction of Nrf2, SFN induces a shift that promotes mitochondrial metabolism and apoptosis and decreases lactate fermentation in prostate cancer cells, an event that seems to sensitize the tumor cell [29]; therefore, SFN is one of the most promising alternatives for treating prostate cancer. The experimental evidence supports a relationship between SFN and a decrease in the expression of HIF-1α and vascular endothelial growth factor (VEGF) in colon cancer [20]. HIF-1α has been associated with resistance to apoptosis, an increase in angiogenesis mediated by VEGF overexpression, and, ultimately, tumor cell metastasis [30].

Several studies have investigated the pharmacological aspects of SFN administered individually and in combination with therapeutic agents, demonstrating that it increases the effectiveness of conventional therapy drugs by exerting a synergistic effect when administered simultaneously [23,31]. Combined treatment of SFN and 5-fluorouracil (5-flu) induces autophagic death in the breast cancer cell line MDA-MB-231, resulting in a significant reduction in the number of live cells compared to treatments alone [32]. In a recent investigation on the combination of SFN and taxanes (Paclitaxel and Docetaxel) in triple-negative breast cancer (TNBC), they suggest synergistic effects to reduce cell line proliferation effectively. In a mouse orthotopic xenograft model with advanced treatment, co-treatment shows a greater reduction in primary tumor volume and significantly reduces secondary tumor formation [23]. However, studies comprising SFN and DCT combinations for prostate cancer treatment are limited. This study aimed to (1) characterize the baseline metabolic profile of the prostate cancer cell lines LNCaP and PC-3; (2) identify changes induced by individual and combined treatments with DCT and SFN on the baseline profile of the cell lines; (3) determine the ability of combined DCT and SFN treatments to induce cell death; and (4) explore potential correlation models between observed changes in metabolic response and cell death.

## 2. Results and Discussion

### 2.1. Baseline Metabolic Characteristics in Prostate Cell Lines

The normal prostate epithelial cell line RWPE-1 and the prostate cancer cell lines LNCaP and PC-3 underwent baseline metabolic characterization before treatment with DCT and SFN. This initial characterization established a reference framework for identifying metabolic changes induced by the subsequent treatments with DCT and SFN (Table 1).

RWPE-1 showed an intermediate amount of remaining glucose (2.98 ± 2.49 pg glucose remaining/µL/cell) compared to LNCaP (4.44 ± 3.78 pg glucose remaining/µL/cell) and PC-3 (0.41 ± 0.39 pg glucose remaining/µL/cell). Regarding lactate production, RWPE-1 also showed moderate levels (0.64 ± 0.95 nmol lactate/L/cell), suggesting lower dependence on aerobic glycolysis. This moderate glucose consumption and lactate production are characteristic of non-cancerous prostatic cells and similar to normal prostatic epithelial cells [33,34].

Surprisingly, ROS production was significantly high in RWPE-1 (431,200 ± 53,204.51 RFI normalized to control) compared to LNCaP and PC-3 cells. This high level of ROS may be related to the high mitochondrial mass observed (1,040,000 ± 38,400 RFI normalized to control), suggesting increased oxidative activity and substantial utilization of oxidative phosphorylation for ATP generation. These findings are consistent with previous research that has established a link between increased ROS production and mitochondrial biogenesis [35]. The moderate expression of *LDHA* in RWPE-1 cells (0.000006 ± 0.000003 relative mRNA expression) further supports the notion that these cells maintain a balance between glycolytic metabolism and oxidative phosphorylation rather than a heavy reliance on glycolysis as is often observed in cancer cells. Respiratory measurements reinforce this hypothesis, with RWPE-1 cells exhibiting basal respiration percentages of 12.20 ± 3.04% OCR, a proton leak of 10.0 ± 2.83% OCR, and a maximal respiratory capacity of 5.35 ± 1.90% OCR. The non-mitochondrial respiration was also low (6.00 ± 0.00% OCR), further indicating a predominantly mitochondrial origin of ATP production, emphasizing an efficient and controlled use of oxidative phosphorylation.

Despite the high ROS production, the GSH levels also indicate that RWPE-1 cells have an efficient antioxidant capacity to neutralize excess ROS. For GSH levels, RWPE-1 showed a mean of 0.16 ± 0.002 umol GSH/µL, reflecting a moderate capacity for detoxification and maintenance of the cellular redox environment. The GSSG level was 0.001879 ± 0.00013 umol GSSG/µL, which, together with GSH, indicates a balanced redox state critical for normal cellular function. The GSH/GSSG ratio was 84.88 ± 4.63, supporting a well-maintained redox environment. The expression of *SOD2*, a marker of oxidative stress and cancer transformation [36], was also low in RWPE-1 (0.0000009 ± 0.0000004 relative mRNA expression), indicating the well-maintained redox state of RWPE-1, necessary for the maintenance of cellular homeostasis.

Compared to RWPE-1, LNCaP cells show the first indications of malignant transformation. LNCaP cells had a significantly higher lactate production (1.39 ± 0.19 nmol lactate/L/cell), suggesting higher glycolytic activity, a characteristic of the Warburg effect [37]. However, the concentration of remaining glucose was high (4.44 ± 3.78 pg remaining glucose/µL/cell), indicating lower glucose consumption. These results may indicate a high conversion of glucose to lactate through aerobic glycolysis, a pathway cancer cells favor to fulfill their energy and biosynthetic requirements, even in the presence of oxygen [38]. The acidic microenvironment caused by high lactate production offers cancer tissues a competitive edge over normal tissues, promoting increased invasion and metastasis [39,40]. Under these acidic conditions, the expression of glucose transporters such as GLUT1 and GLUT4 is often reduced [38], potentially leading to the observed decreased glucose uptake. In the same context, LNCaP cells showed the highest expression of *LDHA* (0.000013 ± 0.000006 mRNA) compared to RWPE-1 and PC-3. Elevated *LDHA* expression is a hallmark of cancer cells as it increases glycolytic flux to support tumors’ high metabolic demands and rapid proliferation [41]. Previous studies have confirmed these findings for LNCaP cells, noting an association with increased glycolytic activity and reduced apoptosis [42]. These results suggest that LNCaP cells have adapted to rely heavily on glycolysis for energy production and to create an environment conducive to tumor survival and growth.

Other data in Table 1 supported the fact that LNCaP cells display a more glycolytic phenotype, such as their lower mitochondrial activity, demonstrated by the low level of basal respiration (8.67 ± 2.52% OCR) in comparison to RWPE-1 and PC-3. Additionally, the proton leak values (21.3 ± 5.86% OCR) suggest inefficiency in ATP production, characteristic of metabolism that does not maximize ATP production through oxidative phosphorylation. Because mitochondrial membrane potential (ΔΨm) reflects the electron transport process that determines oxidative phosphorylation as the driving force for ATP production [43], it is a key indicator of mitochondrial activity. Consequently, a reduced mitochondrial respiratory rate results in a lower ΔΨm, which favors the depletion of ATP production.

Furthermore, the intermediate value of maximal respiratory capacity (8.67 ± 5.51% OCR) indicates a limited capacity to increase ATP production on demand, supporting the idea of reduced reliance on oxidative phosphorylation. Non-mitochondrial respiration in LNCaP cells was also low (6.33 ± 2.52% OCR) compared to PC-3 cells (15.33 ± 3.51% OCR), suggesting that these cells do not significantly rely on non-mitochondrial pathways for energy production. Non-mitochondrial pathways include cytochrome P450 oxidase activity, NADPH oxidase activity, and peroxisomal metabolism that may contribute to oxygen consumption and energy production outside of mitochondria [44,45]. The limited activity of these pathways in LNCaP is in line with their lower aggressiveness and their greater dependence on aerobic glycolysis and reflects the variations in metabolic strategies used by these cells to fulfill their energy requirements and manage oxidative stress [46,47].

LNCaP cells also exhibited moderate GSH levels (0.22 ± 0.045 umol GSH/µL), indicating some antioxidant capacity, although not as high as in PC-3 cells. This could be attributed to their dependence on glycolysis, which generates fewer ROS than oxidative phosphorylation, as evidenced by the lower ROS values reported for LNCaP cells (12,417.5 ± 881.76 RFI normalized to the control). The GSSG levels (0.0026 ± 0.0013 μmol GSSG/µL) were slightly higher than in RWPE-1 and PC-3, suggesting the presence of some oxidative stress. However, the high GSH/GSSG ratio (139.33 ± 50.07) indicates a more balanced redox environment, suggesting lower aggressiveness and the capacity to manage the oxidative stress resulting from glycolytic activity.

When analyzing the glycolytic state, we observed significant statistical differences in the concentration of remaining glucose in PC-3 cells compared to LNCaP (*p* = 0.01) and RWPE-1 (*p* = 0.04). In all cases, PC-3 cells showed much lower remaining glucose levels, indicating a significantly higher rate of glucose consumption. Similarly, PC-3 cells produced significantly less lactate than LNCaP and RWPE-1 cells. This result was unexpected considering the Warburg effect, where an increased lactate production would be expected in cancer cells due to anaerobic glycolysis. However, this view has been challenged by recent investigations [48,49]. Some cancer cells, especially the more aggressive types such as PC-3, can metabolically adapt to use other energy pathways, such as oxidative phosphorylation (OXPHOS) or β-oxidation of fatty acids [50], thereby reducing lactate production [51].

The hypothesis of alternative metabolic adaptations in PC-3 cells was supported by other values shown in Table 1. Compared to LNCaP, PC-3 showed higher mitochondrial mass (160,000 ± 26,519.19 RFI), high basal respiration (29.30 ± 5.03% OCR), and maximal respiratory capacity (16.0 ± 5.29% OCR). In addition, elevated GSH levels (0.37 ± 0.047 μmol/µL) would suggest an increased antioxidant capacity to deal with oxidative stress generated by mitochondrial activity. The low expression of *LDHA* (0.000001 ± 0.0000005 relative mRNA expression) reinforces the idea of a lower conversion of pyruvate to lactate, thus redirecting pyruvate to the Krebs cycle. These results would provide evidence that mitochondrial energy pathways are reprogrammed in highly aggressive cancer cells like PC-3 to address the high energy demand, optimize the use of available fuels, and support macromolecular synthesis necessary for rapid cell division and migration [52].

Next, we identified the changes induced by individual and combined treatments with DCT and SFN on the basal profile; thus, we determined the IC50 of DCT and SFN for each cell line, which allows us to define the specific doses necessary to inhibit cell growth by 50%.

### 2.2. Dose-Response Effects of SFN and DCT Treatments and Combination Analysis

The anti-cancer properties of SFN have been extensively studied and have demonstrated efficacy against several cancers, including prostate cancer. However, one of the most challenging issues with SFN is determining the appropriate dose for in vitro or in vivo models. Our main goal was to avoid using concentrations that were too high or did not represent the true physiological nature of SFN administration. Therefore, we used concentrations corresponding to SFN Cmax from supplementation or broccoli ingestion studies. We maintained low doses for our initial cell viability assays using SFN treatments alone, ensuring a physiologically relevant IC50 (0.5 to 50 µM). Our results correlate with previous SFN administration models used in clinical trials [53,54].

Similarly, the SFN doses used in our cell cultures included Cmax values obtained from patients receiving SFN treatments in breast and prostate cancer chemoprevention trials [55,56]. The importance of our approach translates into better in vivo correlations and avoids misinterpretation of the log concentration in cell culture [57].

**Figure 1:**
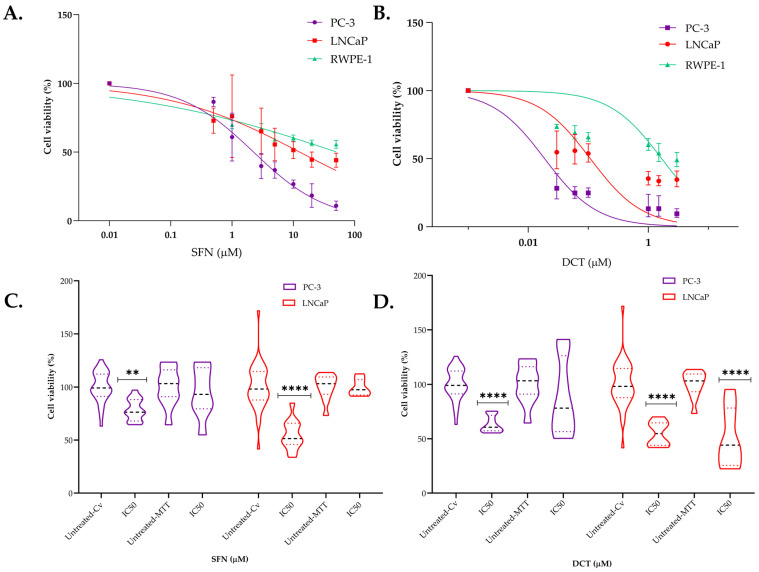
Cell viability and mitochondrial activity induced by SFN and DCT in human prostate cell lines after 48 h of treatment. (**A**) Dose-response curves of non-tumorigenic RWPE-1 epithelial cells and LNCaP and PC-3 prostate cancer cells treated with SFN concentrations (0–50 µM) and (**B**) treated with DCT concentrations (0–3 µM). (**C**) Comparison of cell viability measured by crystal violet assay and mitochondrial activity measured by MTT assay in LNCaP and PC-3 prostate cancer cells treated with the IC50 concentrations of SFN (13.1 and 2.2 µM, respectively) and (**D**) DCT (0.13 and 0.01 µM, respectively) at 48 h. Data represent the mean of n = 3 independent experiments and are presented as mean ± SEM. The dotted line indicates the median value. The values were normalized to the control (untreated cells), and significance is indicated as ** *p* < 0.01 and **** *p* < 0.001.

Our next focus was the combination analysis. First, we analyzed the cell growth inhibitory role of SFN and DCT alone using the RWPE-1, LNCaP, and PC-3 cell lines treated with increasing concentrations of the compounds for 72 h (Supplementary Figure S1). The results showed that both SFN and DCT significantly inhibited the growth of tumor cell lines in a dose- and time-dependent manner in all cell types, with the reduction being particularly pronounced in cancer cells. We chose 48 h treatment as the optimal incubation time to ensure a preferred short exposure time [58]. We analyze whether this 48 h exposure time for SFN and DCT could influence a differential response on RWPE-1 and tumor cells. SFN and DCT IC50 values for RWPE-1 were 40.5 µM and 2.3 µM, respectively. In addition, our model maintained over 50% cell viability in RWPE-1 and reduced cell viability in LNCaP and PC-3. These results demonstrate a higher treatment tolerance of RWPE-1 cells than tumor cells. (Figure 1A,B).

Next, we calculated the 48 h IC50 values for the LNCaP and PC-3 cell lines. The IC50 values for SFN were determined to be 13.1 µM for LNCaP and 2.2 µM for PC-3. For DCT, the IC50 values were 0.1315 µM for LNCaP and 0.01 µM for PC-3. In this study, we used a repeated dose model to simulate administration of our IC50s in both cancer cell lines [59]. Our repeated dose model identified a 2.2 µM SFN concentration for PC-3 cells, which is lower than concentrations reported in previous studies. The discrepancy between our results and those reported in the literature underscores the importance of using repeated dose models to accurately evaluate the effects of SFN on proliferative prostate cancer. Additional in vitro studies using single-dose models for SFN have reported an IC50 of 10 µM for DU145 cell lines at 24 h [60] and a 40–60% reduction in cell viability for DU145 and PC-3 cells following 30 µM SFN administration for 72 h [60]. Our lower IC50 is likely due to the impact of single-dose studies that may mask critical factors such as SFN excretion and the lability of SFN, which can significantly influence the active principle of SFN in highly proliferative prostate cancer cells.

To verify the efficacy of our IC50, we conducted an experimental evaluation to assess cell viability and cytotoxicity in LNCaP and PC-3 tumor cells 48 h post-administration of IC50 SFN and IC50 DCT. The results indicated that both SFN and DCT (Figure 1C,D) individually reduced LNCaP cell viability by approximately 50% (*p* < 0.001) compared to the control. For PC-3 cells, DCT administration also led to a nearly 50% reduction in viability (*p* < 0.001), while SFN treatment maintained 80% cell viability (*p* < 0.01). Our findings suggest that PC-3 is particularly SFN chemo-resistive, indicating possible adaptative responses. This adaptative response might be dose-related since our individual treatments used a 2.2 µM SFN. Previous research has shown that inhibitory concentrations of SFN may need to be higher than 5 µM for PC-3 cells to follow an increased mitochondrial mass after 48 h of exposure [27].

We used MTT reduction assays concomitantly to assess cytotoxicity, as shown in Figure 1C,D. The reduction of MTT happens in metabolically active cells, mainly catalyzed by mitochondrial dehydrogenases, making this measurement an indicator of mitochondrial activity. The results showed that LNCaP and PC-3 cells exposed to SFN did not exhibit significant differences in cell cytotoxicity as measured by the MTT assay. This suggests that the cells conserved mitochondrial activity at our individual IC50s (Figure 1C). As expected, DCT treatment led to a markedly significant reduction in MTT activity in LNCaP compared to PC-3 cells (Figure 1D). MTT results also suggest that the IC50 SFN 48 h dose used in our model did not induce significant cytotoxicity in both cell lines but induced a differential response of DCT, according to the intrinsic cancer cell nature.

To evaluate how SFN and DCT interact with each other in the LNCaP and PC-3 cell lines, specific power relationships were found by slope analysis using non-linear regression. Our initial findings revealed a constant power relationship for SFN and DCT treatments in LNCaP cells, indicating a consistent dose-response effect. However, the situation was more complex in PC-3 cells, where non-constant relationships were observed, leading to different slopes between the dose-response curves (Supplementary Figure S2).

Supplementary Figure S3 illustrates the experimental design for evaluating specific SFN:DCT ratios. In LNCaP cells, the viability curve consistently decreased as the SFN ratio was reduced, particularly at 1:1 and 1:2 ratios. However, in PC-3 cells, a more pronounced decrease in viability was observed at 1:1 and 2:1 ratios, suggesting variability in the response to combination treatments. The 1:1 ratio produced the most significant effect at lower concentrations for the PC-3 cell line, which exhibited signs of chemoresistance. The results of the combination ratios are presented in Supplementary Figure S4.

To address these observations, subsequent assays for both cancer cell lines were conducted using the 1:1 ratio of the obtained IC50 values (1/2 IC50 SFN: 1/2 IC50 DCT). Specifically, 6.55 µM SFN and 0.0675 µM DCT were used for LNCaP cells, and 1.1 µM SFN and 0.0005 µM DCT were used for PC-3 cells, with a 48 h incubation time. The 1:1 ratio allows for the use of lower concentrations of SFN and the chemotherapeutic DCT, making it a desirable treatment condition that minimizes the doses required. All subsequent treatments, including the confirmation of chemosensitization through Annexin V and caspase assays, employed the 1:1 combination for SFN and DCT, as well as the 48 h incubation time.

Testing the 1:1 ratio of SFN and DCT, where each component was used at half its respective IC50 concentration, confirmed its therapeutic potential. This combination reduced cell viability to 68.12% viability in LNCaP cells and 88.12% in PC-3 cells compared to the control (untreated cells) (Supplementary Figure S5). There were no significant differences in cell viability observed in RWPE-1 (non-tumor) cells when evaluating the IC50 concentrations of SFN and DCT found for tumor cells, as well as the 1:1 combination. This finding is particularly significant as it supports the hypothesis that the SFN and DCT combination exhibits selective cytotoxicity towards prostate cancer cells, a potential dose-dependent effect.

Similarly, in prostate cancer, the combination of SFN with taxanes results in increased apoptosis and a reduction in cell proliferation. For instance, in triple-negative breast cancer (TNBC), the combination of SFN with paclitaxel or docetaxel not only enhances the cytotoxicity of bulk tumor cells but also reduces the population of cancer stem cells (CSCs), resulting in a considerable reduction in tumor volume in both in vitro and in vivo models [23]. In pancreatic cancer, the combination of sulforaphane with gemcitabine has been demonstrated to enhance the cytotoxic efficacy and to diminish the colony-forming capacity of cancer cells. Similarly, in prostate cancer, the combination of SFN with taxanes has been demonstrated to increase apoptosis and reduce cell proliferation [23]. These examples demonstrate how SFN can enhance the effects of other drugs, offering a promising approach to improving cancer treatment outcomes.

### 2.3. Effects of Individual and Combined DCT and SFN Treatments on Metabolic Parameters and Redox State in LNCaP and PC-3 Cell Lines

In order to assess the impact of each treatment in LNCaP and PC-3 cancer cell lines, we examined how SFN, DCT, and a combination of SFN and DCT treatments affected different metabolic and oxidative stress factors. These factors included glucose consumption, lactate production, GSH levels, GSH/GSSG ratio, ROS levels, mitochondrial mass, and cellular respiration (Table 2).

The results revealed significant changes in several key parameters following treatments with DCT, SFN, and the SFN:DCT combination in LNCaP and PC-3 cells. LNCaP cells showed a notable increase in ROS levels (1.4793 ± 0.1658) and mitochondrial mass (5.4570 ± 1.4079) under DCT treatment. Similarly, the SFN:DCT combination also increased ROS levels (1.4391 ± 0.0988) and mitochondrial mass (9.1872 ± 2.8282) compared to the control group. These findings suggested that oxidative stress and mitochondrial dysfunction are the observed metabolic changes. Notably, both the DCT and the SFN:DCT combination demonstrated comparable effects. However, the most significant finding was that the SFN:DCT combination achieved these effects at lower doses, using only half of the IC50. This made it a promising option for early-stage prostate cancer treatment, as represented by the LNCaP cell models.

Mitochondrial dysfunction induced by DCT and the SFN:DCT combination treatments in LNCaP cells is supported by other results in Table 2. Under DCT treatment, basal respiration increased to 198.96 ± 39.10% compared to the control (100 ± 27.47%), suggesting elevated mitochondrial activity; this activity could be potentially associated with an effort by the mitochondria to compensate for dysfunction. This increased activity may contribute to ROS production and contribute to oxidative stress [61]. On the other hand, the combined SFN:DCT treatment reduced basal respiration to 66.50 ± 19.52%, indicating a decrease in mitochondrial activity, possibly due to severe mitochondrial damage. Similarly, proton leak was also affected. In control, proton leak was 100%, but under DCT and SFN:DCT combination treatments, this value increased to 307.69 ± 203.26% and 161.54 ± 66.62%, respectively, reflecting a significant loss of mitochondrial efficiency and increased ROS production [62].

Additionally, *SOD2* expression in LNCaP cells treated with DCT decreased dramatically (0.0525 ± 0.0062) compared to the control, which would facilitate ROS accumulation and contribute to oxidative stress and mitochondrial dysfunction. The DCT-SFN combination also reduced *SOD2* expression to 0.1551 ± 0.040, indicating that both treatments decreased antioxidant capacity compared to the control, promoting a prooxidant environment contributing to cell death.

Finally, the treatments with DCT and the SFN:DCT combination induced a prooxidant state that significantly impacted the glycolytic metabolism of LNCaP cells. The observed increase in residual glucose in the medium (from 0.0035 ± 0.0003 pg/μL/cell in the control to 0.1045 ± 0.0034 pg/μL/cell with DCT and 0.0879 ± 0.0061 pg/μL/cell with SFN:DCT) was indicative of a reduction in glucose consumption. Furthermore, the reduction in lactate levels with SFN:DCT (1.2973 ± 0.0856 nmol/L/cell) in comparison to the control (1.5695 ± 0.0021 nmol/L/cell) and the reduced expression of *LDHA* (0.1274 ± 0.1294 with DCT and 0.1953 ± 0.0478 with SFN:DCT) provide additional evidence for the inhibition of glycolysis. These findings are consistent with the hypothesis of a metabolic shift toward OXPHOS, in line with previous studies that have suggested that SFN can induce a similar transition [63].

The behavior of PC-3 cells concerning ROS levels and mitochondrial mass was noteworthy in the context of oxidative stress. The ROS levels decreased from 1.0 ± 0.528 in the control group to 0.3716 ± 0.065 SFN, to 0.4600 ± 0.0192 with SFN:DCT, and to 0.7456 ± 0.0711 with DCT. This reduction in ROS was accompanied by a significant decrease in mitochondrial mass with SFN (0.4925 ± 0.1724), DCT (0.6605 ± 0.1039), and SFN:DCT (0.8903 ± 0.1359) compared to the control group (1.0 ± 0.1637). This suggests that losing dysfunctional mitochondria may reduce ROS production, thereby limiting oxidative stress in PC-3 cells. The potential involvement of mitophagy as a defensive mechanism may elucidate this reduction in mitochondrial mass and its subsequent impact on ROS production. This selective elimination process may also explain the reduction in maximal respiratory capacity. Under DCT treatment, maximal respiratory capacity was reduced to 75.00 ± 28.66%, while with SFN:DCT, it was reduced to 61.36 ± 10.23%, compared to the control (100 ± 17.16%).

The administration of the SFN:DCT combination to PC-3 cells also resulted in a significant reduction in glucose consumption. This change was indicated by the higher residual glucose levels under SFN:DCT (0.0684 ± 0.055 pg/µL/cell) compared to the control (0.0005 ± 0.0001 pg/µL/cell). Moreover, treatment with SFN (0.0752 ± 0.0041 pg/µL/cell) and DCT (0.0690 ± 0.0002 pg/µL/cell) individually also led to a reduction in glycolysis in PC-3 cells. However, despite the decreased glycolytic dependency, lactate production remained high in PC-3 cells treated with the SFN:DCT combination (1.4535 ± 1.1167 nmol/L/cell).

The SFN treatment resulted in a lactate production rate of 1.4391 ± 0.1467 nmol/L/cell, higher than that observed in the control group (0.0955 ± 0.0039 nmol/L/cell). The elevated lactate production rate contrasts with the reduction in *LDHA* expression (0.4789 ± 0.0855) induced by the SFN:DCT combination. These findings suggest that PC-3 cells treated with the combination reduced their direct dependence on glycolysis. However, the stress induced by SFN:DCT does not seem to interfere with alternative metabolic pathways, such as glutaminolysis. Previous literature has indicated the adaptability of androgen-independent cancer cell lines, or resistance phenotypes like PC-3, in their ability to utilize glutamine and increase glutaminolysis as a pathway for lactate synthesis [64,65,66].

The low doses of SFN utilized in the SFN:DCT combination treatment resulted in an unfavorable modulation of the redox response in PC-3. In our study, despite the use of the SFN:DCT combination, an increase in *SOD2* expression was observed, with a value of 5.3560 ± 1.3269 compared to the control group, which exhibited a value of 1.0 ± 0.1175. The administration of SFN resulted in a further increase in *SOD2* expression, reaching a level of 19.7233 ± 3.0496. In contrast, the addition of DCT led to a reduction in this expression to 0.6506 ± 0.1284. Additionally, the GSH levels were elevated in the SFN:DCT group, reaching 0.5952 ± 0.0918 µmoles/µL, in comparison to the control group (0.3685 ± 0.0252 µmoles/µL). The mean GSH levels were 0.5952 ± 0.0918 µmoles/µL for the combination treatment, 0.3339 ± 0.0082 µmoles/µL for DCT, and 0.9673 ± 0.0673 µmoles/µL for SFN.

This resistance in PC-3 cells seems to be mediated by the modulation of antioxidant defenses as an adaptive mechanism to treatment, combined with their glycolytic phenotype and metabolic plasticity. These features probably attenuate the oxidative stress induced by the SFN:DCT combination, thereby limiting its cytotoxicity. Several strategies could be explored to overcome this resistance and increase the efficacy of SFN:DCT in PC-3 cells, including targeting specific metabolic pathways.

One potential target is the ATP-binding cassette transporter G1 (ABCG1), which plays a critical role in cholesterol and lipid efflux [67]. ABCG1 contributes to PC-3 resistance by supporting lipid efflux, metabolic plasticity, and redox balance [68]. Its role in maintaining mitochondrial membrane integrity and reducing oxidative stress is consistent with increased *SOD2* expression and GSH levels in SFN:DCT-treated PC-3 cells. In addition, ABCG1-mediated drug efflux may limit SFN:DCT retention, thereby reducing cytotoxicity [69]. Targeting ABCG1 may enhance the efficacy of the combination by interfering with these adaptive mechanisms [70].

On the other hand, previous research has shown that higher concentrations of SFN (≥5 μM) can enhance chemosensitization in resistant cell lines such as PC-3 and DU145, especially when combined with chemotherapeutic agents such as taxol or cisplatin [71]. Increasing SFN concentrations beyond 5 μM may potentially overcome these compensatory mechanisms in PC-3 cells, as seen at doses such as 10 μM [60]. In addition, the integration of glycolytic inhibitors with the SFN:DCT combination may interfere with the energy supply essential for PC-3 cell survival [72]. Glycolysis inhibition can lead to ATP depletion, triggering apoptosis and overcoming drug resistance by affecting multidrug resistance proteins that require ATP for drug efflux [73,74]. Another viable strategy is disrupting the antioxidant defenses of PC-3 cells, which exhibit robust systems such as elevated thioredoxin reductase activity. Utilizing agents like auranofin, a thioredoxin reductase inhibitor, could increase ROS levels, promoting apoptosis in resistant cancer cells [75,76]. Furthermore, optimizing the ratio of SFN and DCT within the combination therapy could enhance their synergistic potential. Implementation of these strategies could help sensitize PC-3 cells to SFN:DCT therapy, thereby improving treatment outcomes for aggressive and resistant prostate cancer phenotypes.

### 2.4. Effects of Individual and Combined SFN and DCT Treatments on Cell Death

**Figure 2:**
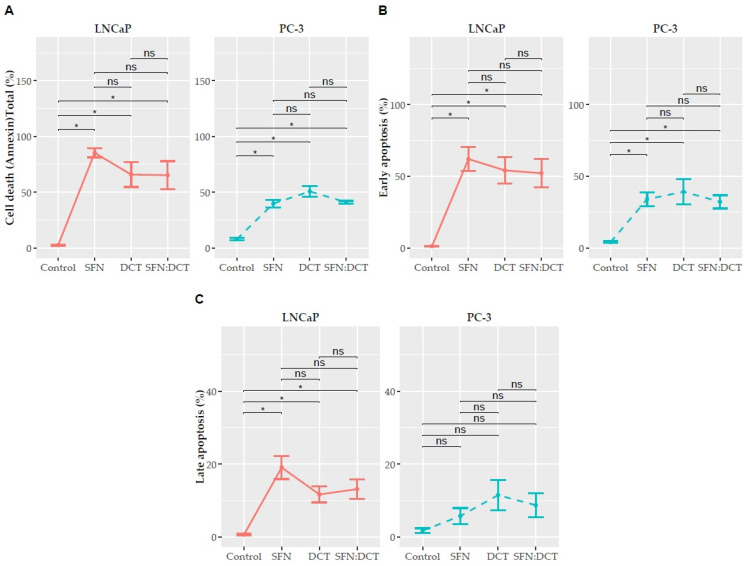
Quantitative analysis of apoptotic effects assessed by Annexin V assay shows the effects of SFN, DCT, and their combination on early and late apoptosis in prostate cancer cells. (**A**) Total cell death: in LNCaP cells, the combination of SFN and DCT induced significantly higher total apoptosis (65.26 ± 35.58%) compared to the control, with no significant difference observed between the single treatments of SFN or DCT. In PC-3 cells, SFN:DCT also showed higher total apoptosis (40.86 ± 30.77%) than control, but no significant difference from DCT alone. (**B**) Early apoptosis: SFN induced the most significant early apoptosis in LNCaP cells (61.99 ± 23.66%), superior to DCT (39.16 ± 25.14%) and SFN:DCT (52.11 ± 28.16%). In PC-3 cells, DCT produced the highest level of early apoptosis (39.16 ± 25.14%), significantly higher than SFN (27.37 ± 8.56%) and SFN:DCT (33.12 ± 22.85%). (**C**) Late apoptosis in LNCaP cells: SFN induced moderate late apoptosis (19.07 ± 8.95%). In PC-3 cells, DCT showed enhanced late apoptosis (24.70 ± 7.12%) compared to SFN (16.49 ± 7.65%) and SFN:DCT (18.75 ± 13.37%). Data are presented as mean ± SEM and represent the average of n = 3 independent experiments. Data were normalized to the control (untreated cells), and statistical significance is indicated as * *p* < 0.05 and not significant (*ns*).

**Figure 3:**
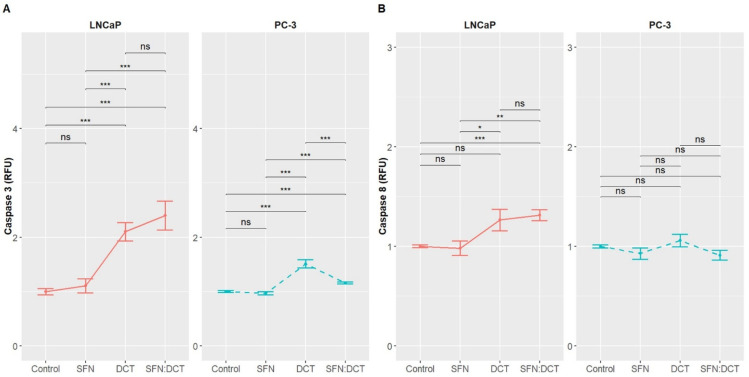
Induction of Apoptosis Mediated by Activation of Caspases 3 and 8 in LNCaP and PC-3 Cells. (**A**) The SFN:DCT combination significantly increased the activation of LNCaP cells (2.4 ± 0.75 RFU) compared with DCT (2.1 ± 0.47 RFU) and SFN (1.1 ± 0.37 RFU), respectively. For PC-3 cells, the SFN:DCT combination showed significant activation (1.16 ± 0.0484 RFU), similar to the DCT treatment (1.51 ± 0.2062 RFU), but significantly higher than SFN (0.97 ± 0.0837 RFU) and the control group. (**B**) The combination of SFN and DCT resulted in a statistically significant activation of caspase 8, with a mean value of 1.31 ± 0.15 RFU compared to the control group. However, no significant differences in caspase 8 activation were observed between the individual treatments of SFN and DCT. In PC-3 cells, no statistically significant differences in caspase 8 activation were observed with any of the evaluated treatments. Data represent the mean of n = 3 independent experiments and are presented as mean ± SEM. The data were normalized to the control (untreated cells), and statistical significance is indicated as * *p* < 0.05, ** *p* < 0.01, *** *p* < 0.001, and not significant (ns).

Our previous analyses provided a comprehensive overview of the functional alterations induced by SFN, DCT, and SFN:DCT treatments, revealing changes in energy metabolism and oxidative status that predispose cells to stress and dysfunction. However, to comprehend the efficacy of these treatments in a therapeutic context, we determined whether the observed alterations were associated with apoptosis and, consequently, with cell death by employing an Annexin V assay (Figure 2); to further elucidate the mechanisms driving these apoptotic processes, we measured the levels of caspases 3 and 8 as crucial indicators of the activation of intrinsic and extrinsic apoptotic pathways, respectively (Figure 3). This approach allowed us to better understand the underlying mechanisms driving these outcomes.

A beta regression was employed to quantify the impact of the treatments on apoptosis in the LNCaP (Table 3) and PC-3 (Table 4) cell lines. The beta coefficients were used to measure the magnitude of the effect of each treatment, allowing us to compare their relative efficacy in inducing early apoptosis, late apoptosis, and total cell death. Subsequently, we assessed which parameters contributed to the observed effect on cell death.

In LNCaP cells, treatments with DCT, SFN, and the SFN:DCT combination significantly influenced early and late apoptosis, as shown by the beta coefficients and *p*-values in Table 4. Beta coefficients for early apoptosis were highest for SFN (β = 3.3, 95% CI: 1.9–4.6, *p* < 0.001), followed by DCT (β = 2.9, 95% CI: 1.6–4.3, *p* < 0.001) and the SFN:DCT combination (β = 2.9, 95% CI: 1.3–4.2, *p* < 0.001). Interestingly, while the percentages of early apoptosis were slightly higher in SFN alone compared to the SFN:DCT combination, this difference was not statistically significant (Figure 2B,C). This suggests that SFN alone may exert a stronger apoptotic effect under certain conditions; however, the SFN:DCT combination achieves comparable efficacy at lower doses, thereby reducing potential toxicity.

For late apoptosis, the beta coefficients were also highest in SFN (β = 2.7, 95% CI: 1.7–3.7, *p* < 0.001), followed by the SFN:DCT combination (β = 2.2, 95% CI: 1.2–3.3, *p* < 0.001) and DCT (β = 2.1, 95% CI: 1.1–3.2, *p* < 0.001). Again, the percentages of late apoptosis were slightly higher in SFN alone compared to the SFN:DCT combination, yet the latter maintained therapeutic efficacy while minimizing dosage and associated side effects. These results highlight that although SFN alone may induce stronger apoptotic responses, the SFN:DCT combination offers a balanced approach by maintaining efficacy and reducing toxicity, making it a promising strategy for early-stage prostate cancer.

In the beta regression model, additional parameters, including remaining glucose and mitochondrial mass, were associated with cell death in LNCaP. The beta coefficients for glucose were elevated for both early and late apoptosis, with values of 50 (95% CI: 38–62, *p* < 0.001) and 22 (95% CI: 12–32, *p* < 0.001), respectively. This indicates that reduced glucose consumption is strongly correlated with the induction of apoptosis in LNCaP. It may be inferred that under treatment, LNCaP cells are shifting towards an alternative energy dependence, such as oxidative phosphorylation. This is further supported by the substantial impact of mitochondrial mass on initiating all stages of apoptosis. The beta coefficients indicated a significant impact of this parameter on both early apoptosis (β = 0.28, 95% CI: 0.14–0.42, *p* < 0.001) and late apoptosis (β = 0.24, 95% CI: 0.11–0.36, *p* < 0.001). These findings support prior observations that oxidative stress and mitochondrial dysfunction are closely associated with the induction of cell death in LNCaP cells exposed to SFN and combinations with SFN.

A comparison of these findings with those presented in Figure 3 revealed that the SFN:DCT combination resulted in the most pronounced elevation in caspase activity, particularly caspase 3, with a statistically significant increase (2.4 ± 0.75 RFU) relative to the control (1.0 ± 0.16 RFU). This indicates a collaborative effect between the two compounds in promoting apoptosis in LNCaP cells. This also reinforces the notion that the combination affects cellular metabolism, as postulated by the beta regression, and enhances the intrinsic apoptosis pathway, primarily mediated by caspase 3 activation [77]. While caspase 8 activity was also observed to be activated under the SFN:DCT combination treatment (1.31 ± 0.15 RFU), its activation was less pronounced, indicating that this treatment relies to a greater extent on the intrinsic pathway for apoptosis induction.

**Figure 4:**
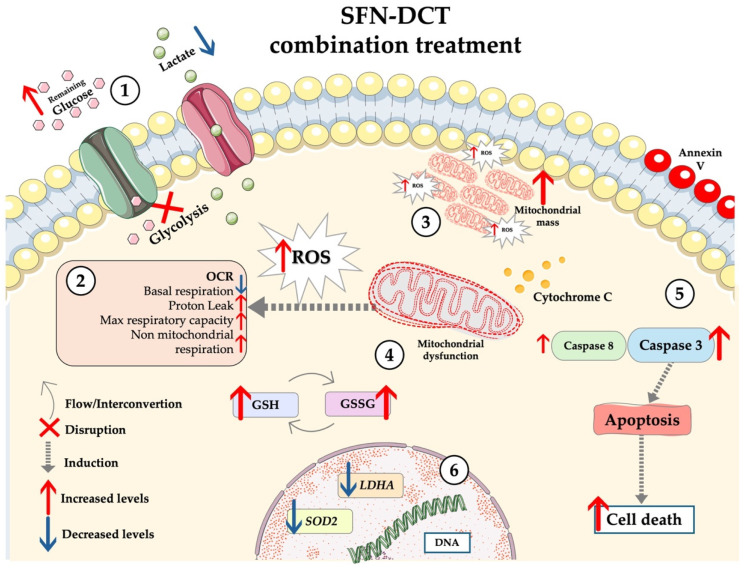
Graphical model describing the mechanism induced by the SFT-DCT combination in LNCaP cells. The SFN-DCT combination induces metabolic and redox changes in treated cells that include (1) Decreased glucose consumption, reflected by elevated residual glucose levels in the medium, and reduced lactate production, indicating a metabolic shift away from glycolysis; (2) The combined SFN:DCT treatment reduced basal respiration, indicating a decrease in mitochondrial activity, possibly due to severe mitochondrial damage. Similarly, proton leak was also affected, reflecting a significant loss of mitochondrial efficiency; (3) Mitochondrial mass increased, suggesting compensatory mitochondrial biogenesis in response to damage, which in turn contributes to the generation of additional ROS; (4) Elevated ROS levels result from mitochondrial dysfunction, overwhelming the antioxidant defense mechanisms, including GSH/GSSG systems. These redox imbalances create a prooxidant environment conducive to cell damage; (5) The increase in ROS production and mitochondrial dysfunction likely triggers the release of cytochrome C, which promotes the activation of caspases 3 and 8. Notably, the higher expression of caspase 3 suggests the activation of the intrinsic apoptotic pathway leading to cell death, as confirmed by Annexin V; (6) While oxidative stress is evident, the combination also reduces *LDHA* expression, further inhibiting glycolysis. Decreased *SOD2* expression reflects weakened antioxidant capacity, exacerbating oxidative stress and reinforcing the apoptotic processes triggered by the SFN:DCT combination.

Figure 4 provides a comprehensive overview of the effects of SFN:DCT treatment on LNCaP cells, highlighting key metabolic and redox changes.

In PC-3 cells, the activation of apoptotic processes under DCT, SFN, and SFN:DCT treatments showed notable differences compared to LNCaP cells (Figure 2). Firstly, caspase 3 activation in PC-3 was significantly lower than in LNCaP, with levels of 1.51 ± 0.2062 RFU for DCT and 1.16 ± 0.0484 RFU for the SFN:DCT combination (Figure 3), compared to the higher values observed in LNCaP (2.1 ± 0.47 and 2.4 ± 0.75 RFU, respectively). This may suggest that although the intrinsic pathway is activated in PC-3, the apoptotic response is less pronounced than in LNCaP. Similarly, caspase 8 showed much lower activation in PC-3 (DCT: 1.06 ± 0.1763 RFU; SFN:DCT: 0.91 ± 0.1384 RFU) compared to LNCaP, where DCT reached 1.26 ± 0.31 RFU and SFN:DCT 1.31 ± 0.15 RFU, suggesting that the extrinsic apoptosis pathway is also less active in PC-3. These differences highlight the greater resistance to apoptosis in PC-3 cells, likely due to their more metastatic and aggressive nature, making them less susceptible to the activation of apoptotic pathways than LNCaP cells.

Similarly, in the regression model, treatments with DCT, SFN, and the SFN:DCT combination showed significant effects on the induction of early apoptosis; however, none clearly impacted late apoptosis, as observed in the beta coefficients and *p*-values (Table 4). The DCT treatment was the most effective in inducing early apoptosis, with a beta coefficient of 2.1 (95% CI: 0.99–3.1, *p* < 0.001), followed by SFN (1.9, 95% CI: 0.85–3.0, *p* < 0.001) and the SFN:DCT combination (1.8, 95% CI: 0.77–2.9, *p* < 0.001). Despite these values, none of the treatments significantly affected late apoptosis, where the beta coefficients were also close to zero (DCT: β = 0.79, *p* = 0.2; SFN: β = 0.28, *p* = 0.7; SFN:DCT: β = 0.50, *p* = 0.4), indicating that the resistance of PC-3 cells to cell death is maintained at more advanced stages of the apoptotic process.

In the beta model, several factors appeared to influence cell death in PC-3, with some differences compared to LNCaP. The remaining glucose showed a high beta coefficient for early apoptosis (β = 30, 95% CI: 12–47, *p* < 0.001), indicating that reduced glucose consumption was strongly associated with the induction of apoptosis in these cells. Glucose appears to be a key factor in the resistance of PC-3, which is more glycolytic, and its reduction directly contributes to the activation of apoptosis. Lactate also showed a significant increase (β = 1.6, 95% CI: 0.66–2.5, *p* < 0.001), indicative of changes in anaerobic metabolism.

With respect to mitochondrial mass, the negative beta coefficients observed during the early phase of apoptosis (β = −2.0, 95% CI: −3.6 to −0.49, *p* = 0.010) imply that a decline in mitochondrial mass is concomitant with the induction of apoptosis. This reduction in mitochondrial mass may be related to mitophagy processes, whereby cells remove damaged mitochondria in order to prevent an excess of oxidative stress. This strategy may account for the observed reduction in ROS accumulation in PC-3 cells, as reflected in the negative beta values for ROS (β = −2.2, 95% CI: −3.9 to −0.47, *p* = 0.012). It is possible that these cells are utilizing alternative pathways to maintain their survival, in contrast to LNCaP cells, where mitochondrial stress was higher. Prior research has demonstrated that SFN induces autophagy in prostate cancer cells at elevated concentrations and over extended treatment periods [25,78]. However, the same authors have previously proposed this as a potential defensive mechanism against SFN-induced apoptosis at lower concentrations in prostate cancer [25]. It is therefore proposed that autophagy plays a role in the sequestration of mitochondria within autophagosomes, which results in the delayed release of cytochrome c and subsequent evasion and/or activation of the caspase cascade. The autophagic defense theory is corroborated by other findings described above, such as the reduction in basal respiration with DCT (73.15 ± 44.17%) and with SFN:DCT (59.26%). This is evidenced by the observation that the respiratory function of mitochondria is compromised, as indicated by a reduction in rates of OCR compared to control samples (100 ± 30.32%), with a decrease of ±9.80% (versus control). This finding is consistent with the elimination of damaged mitochondria through mitophagy.

**Figure 5:**
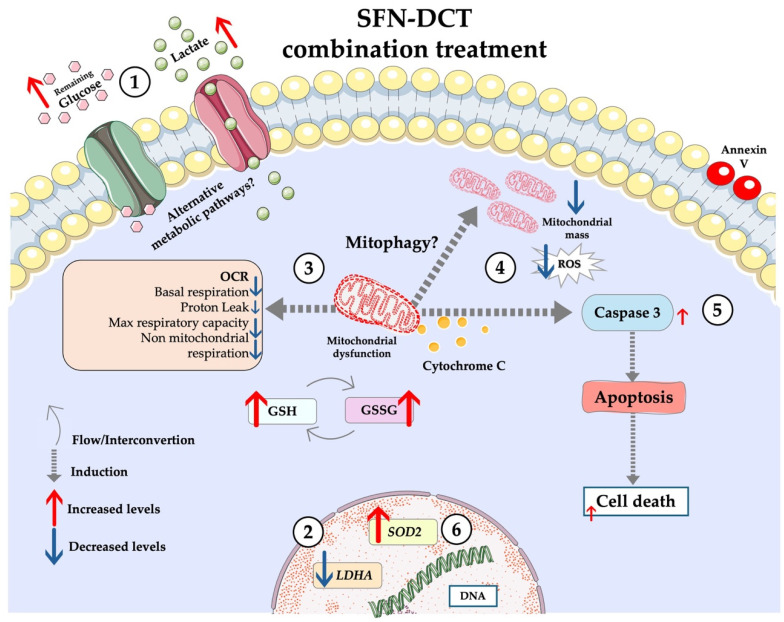
Graphical model describing the mechanism induced by the SFT-DCT combination in PC-3 cells. The SFN-DCT combination induced distinct metabolic and redox responses in PC-3 cells, reflecting their resistance mechanisms: (1) Glucose consumption was reduced, as shown by increased residual glucose levels, while lactate production remained consistent, suggesting a compensatory reliance on alternative metabolic pathways; (2) *LDHA* expression decreased, indicating a shift away from glycolysis as the primary energy source; (3) Key respiratory parameters, including basal respiration, proton leak, and maximal respiratory capacity, were diminished, highlighting mitochondrial dysfunction as a consequence of SFN:DCT treatment; (4) This mitochondrial dysfunction was accompanied by a reduction in ROS levels and mitochondrial mass, suggesting that mitophagy may be actively eliminating damaged mitochondria to maintain cellular homeostasis; (5) Despite the mitophagic response, the activation of caspase 3 demonstrates that the intrinsic apoptotic pathway is triggered, ultimately leading to a modest induction of cell death, confirmed by a moderate Annexin V detection, underscoring the limited cytotoxic impact of SFN:DCT in PC-3 cells compared to more sensitive cell lines like LNCaP; (6) Additionally, oxidative stress markers revealed elevated PC-3 antioxidant defenses, with increased *SOD2* expression and GSH levels, which likely counteract the oxidative damage induced by the SFN:DCT combination. These defenses limit ROS accumulation, contributing to the cells’ resistance mechanisms.

The mitophagy mechanism enables PC-3 cells to maintain resistance to apoptosis. Mitochondrial dysfunction resulting from mitophagy is linked to reduced OXPHOS activity and deregulated ROS production. These factors have been linked to increased prostate cancer progression. However, our findings indicate that despite a potential initial mitophagic event, the reduction in mitochondrial mass may not be sufficient to prevent apoptotic processes. This phenomenon, where mitophagy can lead to cell death, has been previously documented in prolonged damage-related processes [79]. Other chemoresistance mechanisms of PC-3 cells can be related to increased ABCG1 expression [80,81]. Figure 5 summarizes the effects of SFN:DCT treatment on PC-3 cells, highlighting metabolic adaptations, mitochondrial changes, and apoptosis induction.

Despite the strengths and findings of this study, certain limitations must be acknowledged to provide a balanced interpretation of the results. While this study demonstrates that the SFN:DCT combination induces intrinsic apoptosis in prostate cancer cell lines, certain limitations should be acknowledged. Although caspase 3 activation provides evidence for the involvement of the intrinsic apoptotic pathway, additional markers such as cytochrome c, caspase 9, BID, BAX, or cleaved PARP were not assessed. These markers could provide a more comprehensive understanding of the molecular mechanisms driving apoptosis, particularly the role of mitochondrial dysfunction. Their inclusion would strengthen the interpretation of the findings, especially regarding the differences in apoptotic responses between LNCaP and PC-3 cells. Future studies should incorporate these analyses to confirm the involvement of the intrinsic pathway and further elucidate the metabolic and apoptotic vulnerabilities targeted by SFN:DCT.

## 3. Materials and Methods

### 3.1. Cell Cultures

Human prostate cancer cell lines LNCaP and PC-3, and the immortalized non-cancerous prostate epithelial cell line RWPE-1, were purchased from the American Type Culture Collection (ATCC, Manassas, VA, USA). Our metabolic-based experiments focused on how differences in androgen receptor (AR) expression might influence the biological response of our proposed treatments. Therefore, we used RWPE-1 and LNCaP cells with PSA and AR expression that show androgen-mediated growth stimulation. In contrast, PC-3 cells do not express AR and PSA, and their proliferation is androgen-independent. The RWPE-1 cell line was cultivated in keratinocyte serum-free medium (K-SFM) (Thermo Fisher Scientific, Waltham, MA, USA), which was supplemented with bovine pituitary extract (BPE; 0.05 mg/mL) and epidermal growth factor (EGF; 5 ng/mL) and a 1% antibiotic cocktail (penicillin/streptomycin) (Sigma-Aldrich, St. Louis, MO, USA) [82]. In contrast, LNCaP and PC-3 cell lines were cultured in Advanced RPMI 1640 medium (Gibco-Thermo Fisher Scientific, Gaithersburg, MD, USA), supplemented with 10% fetal bovine serum (FBS) (Gibco, Grand Island, NY, USA), and 1% antibiotic cocktail (penicillin/streptomycin), and incubated with 95% air—5% CO_2_ at 37 °C, humidity ≥ 95%. Cells were subcultured and used for assays at approximately 80% confluence.

### 3.2. Treatments

For treatments, D-L sulforaphane ≥ 90% HPLC grade (S4441-5MG, Sigma-Aldrich, St. Louis, MO, USA) was used as a 100 mM stock solution in DMSO. From this stock, a working solution of 100 μM SFN was prepared in RPMI simple media. Then, serial dilutions were prepared for each final tested SFN concentration at sterile conditions.

As commercially available Taxotere^®^, docetaxel was purchased from Aventis Pharmaceutical as a 20 mg/mL vial (Sanofi-Aventis Deutschland GmbH, Frankfurt, Germany), provided directly from Liga Colombiana contra el Cancer. First, a 100 μM DCT working solution was prepared using RPMI simple media for dilution. Then, serial dilutions were prepared for each final tested DCT concentration at sterile conditions. Evaluated SFN concentrations were 0.5, 1, 3, 5, 10, 20, and 50 μM, whereas DCT concentrations were 0.03, 0.06, 0.1, 1, 1.5, and 3 μM.

### 3.3. Cell Viability Assay

The cell viability assay as crystal violet staining measured baseline IC50 (mean inhibitory concentration 50) in all cultured cell lines. Briefly, RWPE-1, LNCaP, and PC-3 cells were seeded in 96-well microplates at a density of 5 × 10^3^ cells/well, allowing them to adhere and proliferate for 24 h. Next, cells were incubated in freshly replaced media containing SFN and DCT. Every 24 h, culture media was replaced to establish dose-response relationships. Therefore, cell viability assay was performed at 24, 48, and 72 h post-treatment. At the end of each period, media was extracted for metabolic measurements (glucose and lactate contents). Obtained cells were washed with phosphate-buffered saline (PBS 1X) (Sigma-Aldrich, St. Louis, MO, USA), fixed with 100 μL of 4% paraformaldehyde (Sigma-Aldrich, St. Louis, MO, USA) for 30 min per well at room temperature, and finally with 100 μL of 0.5% crystal violet solution in 6% methanol (Sigma-Aldrich, St. Louis, MO, USA) at the same time and temperature conditions as before. Microplates containing crystal violet were carefully washed with distilled water for dye remotion, air dried, and protected from light for 24 h. Methanol (200 μL/well) was used to extract the crystal violet bound to the DNA. Microplates were incubated for 20 min at room temperature on an orbital shaker with a frequency of 20 oscillations per minute. Finally, absorbance was determined at an optical density of 570 nm using an Epoch 2 microplate reader (BioTek Instruments, Winooski, VT, USA) using Gen5^®^ Microplate Reader software V3.05.11, BioTek Instruments, Inc., Winooski, VT, USA). Untreated cells were used as a negative control, and 0.4% sodium dodecyl sulfate (SDS) solution (Sigma-Aldrich, St. Louis, MO, USA) was used as a positive control [83].

The absorbance values were normalized by considering 100% of the absorbance obtained from untreated cultures. The results were expressed as dose-response curves. IC50 values were calculated for all cell lines from the steepness (R^2^ > 0.95) of the Hill slope curve for all experimental data using nonlinear regression analysis in GraphPad Prism Software version 9. The results of cell viability experiments are summarized in Supplementary Figure S1.

We utilized a repeated dose model for all cell viability experiments conducted in this study. In this model, we ensured a cell media replacement after each 24 h exposure to SFN, DCT, or a combination of SFN and DCT. Following each treatment, absorbance was measured during each incubation time (24, 48, and 72 h), and we optimized experiments to 48 h treatment using previous cell viability data. This regular media refresh mimics drug administration, minimizing the impact of cell media, reducing SFN lability, ensuring larger bioaccumulation, and allowing the assessment of long-term administration effects [84].

### 3.4. Cytotoxicity Assay

The effect of SFN and DCT on cytotoxicity was determined by the MTT (3-(4,5-dimethylthiazol-2-yl)-2,5-diphenyltetrazolium bromide) assay (Sigma-Aldrich, St. Louis, MO, USA. For this purpose, RWPE-1, LNCaP, and PC-3 cells were seeded in 96-well microplates at a density of 5 × 10^3^ cells/well, allowing them to adhere and proliferate for 24 h. After this, the cells were incubated in media containing the previously calculated IC50 of SFN and DCT (concentrations previously determined by dose-response curves). At the end of the incubation period, the treatments were removed, and 100 μL/well of MTT) was added at a concentration of 0.125 mg/mL; the plate was incubated at 5% CO_2_ at 37 °C for 4 h. Subsequently, the MTT was discarded, and the formazan crystals deposited at the bottom of each well were dissolved in 100 μL of dimethyl sulfoxide (DMSO) (Sigma-Aldrich, St. Louis, MO, USA). Finally, absorbance was determined as mentioned above, and the obtained data were normalized considering 100% of the absorbance obtained from untreated cultures [85,86].

### 3.5. Combination Assays

The experimental design for combination analysis included using the crystal violet assay to test defined combinations of SFN and DCT in the LNCaP and PC-3 cell lines. First, the experimental IC50 was determined for SFN and DCT individually for each prostate cancer cell line and incubation time (see Supplementary Table S1). The following assays used the optimal incubation time and the same initial SFN and DCT concentrations to assess cell viability for each cancer cell line and to analyze the potency relationships established for each substance (see Supplementary Figure S2). Potency relationships allow us to infer whether the combined dose relationships of SFN or DCT produce the same effect as individual treatments.

To further test combinations, we designed crystal violet experiments using IC50-based 6-point combination ratios of 1:1 (1/2 IC50 SFN: 1/2 IC50 DCT), 1:2 (1/2 IC50 SFN: 2 IC50 DCT), and 2:1 (2 IC50 SFN: 1/2 IC50 DCT) (see Supplementary Figure S4). The experiment rationale is specified in Supplementary Figure S3. Briefly, each combination ratio used either a decremental or incremental concentration. For 1/2 IC50s, the combined treatment used a decremental half of the IC50 value, and so on for each of the 6-point cell viability curves. For 2 IC50, the combined treatment used an incremental 2-fold concentration in the combined dose.

The obtained dose-response curves were analyzed to determine the optimal combination ratio using the IC50 curves and potency relationships determined for each cell line. Annexin V and caspase assays confirmed the chemo-sensitization of LNCaP and PC-3 cells in the optimal SFN:DCT combination ratio.

### 3.6. Detection and Apoptosis Characterization

LNCaP and PC-3 cells were previously treated with individual IC50 and the best SFN:DCT combination ratio for analysis for early/late apoptotic cell events using a Muse™ Cell Analyzer (Millipore Corp., Hayward, CA, USA) and the Muse™ Annexin V and Dead Cell Assay (MCH100105, Luminex Corporation, Austin, TX, USA) accordingly to the manufacturer’s instructions. Briefly, 100 µL of cell extract was used to determine the frequency of live, early apoptotic, late apoptotic, total apoptotic, and dead cells. Results were expressed as a percentage of apoptotic cells. Untreated cells were used as the negative control.

To gain insight into the mechanisms involved in the induction of death of prostate cancer cells treated with SFN and DCT, LNCaP and PC-3 cells were seeded at a density of 2 × 10^4^ cells/well in 96-well black-walled, transparent-bottom microplates (Corning Glass, Corning, NY, USA) and treated with the previously determined IC50 of SFN and DCT for 48 h incubation. For each cell line, a control group and a group treated with SFN and DCT were maintained. After the treatments, the induction of apoptosis was assessed using the Caspase 3, Caspase 8, and Caspase 9 Multiplex Activity Assay Kit Fluorometric (ab219915, Abcam, Cambridge, UK) following the manufacturer’s instructions. Briefly, 100 µL/well of the caspase assay loading solution was added directly to the cell plate without removing the culture medium with the treatment solution. Next, the plate was incubated at room temperature for 60 min, protected from light. Fluorescence enhancement was measured in a CLARIOstar^®^ fluorescence microplate reader (BMG Labtech, Ortenberg, Germany) at the specific wavelengths: Caspase 3: Ex/Em = 535/620 nm (red); Caspase 8: Ex/Em = 490/525 nm (green); and Caspase 9: Ex/Em = 370/450 nm (blue). Results were expressed as Relative Fluorescent Units (RFU).

### 3.7. Characterization of the Effects of Combined Treatments in Metabolic and Redox Status of the Prostate Cancer Model

LNCaP and PC-3 cells, as well as RWPE-1 cells, were seeded at a density of 1.2 × 10^6^ cells in 75 cm^2^ culture flasks and harvested for the determination of intracellular levels of reactive oxygen species (ROS), relative mitochondrial mass [27], and the reduced glutathione (GSH)/oxidized glutathione (GSSG) ratio. In addition, the remaining culture media was used to measure glucose and lactate contents to estimate the glycolytic rate. All determinations were performed over basal cultured RWPE-1, LNCaP, and PC-3 cells. Tumor cells treated for 48 h were also analyzed. Cells were treated with individual SFN and DCT concentrations and the optimal SFN:DCT combination ratio.

### 3.8. Determination of Intracellular ROS

Intracellular levels of ROS were determined by using an ROS oxygen species detection reagent (Molecular Probes, Eugene, OR, USA) containing the fluorescent probe 5-(y-6)-chloromethyl-2′7′-dichlorodihydroflourescein diacetate (CM-H_2_DCFDA). The increase in fluorescence due to the oxidation of the probe is detected by flow cytometry. Following SFN and DCT treatments, the cells were dissociated and suspended in 500 µL of culture medium at 2.75 µM of CM-H_2_DCFDA probe, and the cells were incubated for 45 min protected from light at 37 °C. Subsequently, they were centrifuged, the medium containing the probe was removed, and the cells were suspended in 400 µL of 1X PBS. Samples were analyzed on a flow cytometer, Cytek Northern Lights-CLC^®^ (Biosciences, Fremont, CA, USA), the acquired data were analyzed using FlowJo™ V.10.8.1 (BD Life Sciences, Ashland, Oregon, USA) to calculate the average fluorescence intensity per cell, reflecting the relative level of ROS. Fluorescence was measured at a wavelength of Ex/Em: ∼492–495/517–527 nm, respectively. Results were expressed as Relative Fluorescence Intensity (RFI) non-normalized data.

### 3.9. Relative Mitochondrial Mass

Relative mitochondrial mass was determined by staining SFN and DCT-treated cells at the end of treatment. For this purpose, mitochondria were labeled by incubating the cells with a MitoTracker Green FM probe (M7514 Invitrogen™, Eugene, OR, USA), which passively diffuses across the plasma membrane and accumulates in active mitochondria [27]. First, cells were dissociated with Triple Express (1X) (Thermo Fisher Scientific, Carlsbad, CA, USA), centrifuged, and suspended in 500 μL of fresh culture media solution with 150 nM MitoTracker Green FM incubated and protected from light for 45 min at 37 °C. Cells were then centrifuged, suspended in 1X PBS, and analyzed on a flow cytometer, Cytek Northern Lights-CLC^®^ (Cytek Biosciences, Fremont, CA, USA), the acquired data were analyzed using FlowJo™ V.10.8.1 (BD Life Sciences, Ashland, OR, USA), with the appropriate channels configured for MitoTracker^®^ Green FM (Ex/Em = 490/516 nm, green). Results were reported as normalized Relative Fluorescence Intensity (RFI) values. 

### 3.10. Determination of GSH, GSSG, and GSH/GSSG Ratio

The quantification kit for oxidized and reduced glutathione (38185 Sigma-Aldrich, St. Louis, MO, USA) was used according to the manufacturer’s instructions. Glutathione is generally present as a reduced form (GSH) at the cellular level, but GSH is converted to an oxidized form (GSSG) by stimulating oxidative stress. Untreated (basal) cells and SFN, DCT, and SFN:DCT-treated cells were isolated and pretreated according to the protocol. Spectrometric determination was performed by measuring the absorption at 412 nm, derived from a colorimetric reaction of DTNB (5,5′-dithiobis (2-nitrobenzoic acid)) together with enzymatic recycling. Absorbance reading was performed using an Epoch 2 microplate reader (BioTek Instruments, Winooski, VT, USA) at 412 nm (endpoint reading). Results were expressed as μmol/μL. In addition, the GSH/GSSG ratio was calculated as an index of cellular oxidative stress.

### 3.11. Glucose Consumption and Lactate Production Determination

Basal measurements of glucose and lactate production were measured at 24, 48, and 72 h. The supernatant (culture medium of control cells as well as SFN- and DCT-treated cells were collected during the treatment time and deproteinized using the Deproteinization-TCA kit (ab204708, Abcam, Cambridge, UK) and Halt™ protease inhibition cocktail, EDTA-free (Thermo Fisher Scientific, Rockford, IL, USA). Samples were stored at −20 °C until use.

The lactate level at the end of the incubation period was determined using the MAK064 lactate assay kit (Sigma-Aldrich, St. Louis, MO, USA). For this, a colorimetric lactate detection standard curve was constructed in a 96-well plate, generating standards of 0 (blank), 2, 4, 6, 8, and 10 nmoles/well, and lactate assay buffer was added to each well to bring the volume to 50 µL. Approximately 50 µL of sample/well was used, and then 50 µL of the lactate Master Reaction Mix was added to each of the wells. Mixing was carried out using a horizontal shaker; the reaction was incubated for 30 min at room temperature and protected from light. The absorbance was measured at 570 nm in an Epoch 2 microplate reader (BioTek Instruments, Winooski, VT, USA). The results are expressed as nmoles/µL of lactate; the data were normalized considering the concentration obtained from untreated cultures.

The initial glucose levels and remaining glucose in the culture medium for obtaining cellular glucose consumption were determined using the GAGO20 glucose assay kit (Sigma-Aldrich, St. Louis, MO, USA), following the manufacturer’s instructions, miniaturized for 96-well plates. A standard glucose colorimetric detection curve of 20–80 g/µL was constructed. Once 40 µL of the previously treated samples were added, 80 µL of the test reagent was added and mixed for 1 min using an orbital shaker, and the reaction was incubated for 30 min at a temperature of 37 °C. After the time elapsed, the reaction was stopped by adding 80 µL of 12 N sulfuric acid to each well. Each well was carefully mixed, and the absorbance of each well was measured at 540 nm in an Epoch 2 microplate reader (BioTek Instruments, Winooski, VT, USA). The results were expressed as µg/µL of glucose; the data were normalized considering the concentration obtained from untreated cultures.

### 3.12. Expression Levels (mRNA) of SOD2 and LDHA Gene Expression

In order to analyze the gene expression profiles of *SOD2* and *LDHA* (Nrf2 and HIF-1 α-regulated genes) in prostate cancer, the differential basal state expression of the aforementioned genes was evaluated for each cell lineage. For this purpose, RWPE-1, LNCaP, and PC-3 cells were seeded at a density of 1.2 × 10^6^ cells in 75 cm^2^ culture flasks and harvested after 48 h of SFN, DCT, and SFN:DCT treatments. Total RNA extraction was performed using the PureLink RNA Mini kit (12183025, Invitrogen™, Carlsbad, CA, USA according to the manufacturer’s instructions. cDNA was prepared from 1 µg of RNA using a High-Capacity cDNA Reverse Transcription Kit (Thermo Fisher Scientific, London, UK). Expression assays were performed with TaqMan^®^ probes, SOD2 Dye: FAM-MGB (ID: Hs00167309_m1) y LDHA Dye: FAM-MGB (ID: Hs01378790_g1) using the quantitative polymerase chain reaction method of quantitative RT-PCR. In addition, the 18S probe Dye: VIC-MGB (ID: Hs03003631_g1) was used as an endogenous control. Interest probes and endogenous control were mounted as triplicates for each sample. Quantification was performed on the Cobas^®^ Z 480 system (Roche Diagnostics, Rotkreuz, Switzerland) and Light Cycler^®^ Systems Training software (Expert Level) using the relative quantification (RQ) method [87,88].

### 3.13. Respirometry Analysis

Cellular respiration of RWPE-1, LNCaP, and PC-3 cells was measured under basal conditions in a high-resolution Oxygraph-2k respirometer (Oroboros Instruments, Innsbruck, Austria) using two chambers at 37 °C with gentle agitation. Cells under SFN, DCT, and SFN:DCT treatments were also analyzed. The respirometry assay was performed using the sequential addition of mitochondrial stressors: oxidative phosphorylation inhibitor oligomycin (2.2 μM), followed by the mitochondrial uncoupler carbonyl cyanide 4-(trifluoromethoxy)-phenylhydrazone (FCCP; at 0.75 μM for PC-3 cells and 0.5 μM for LNCaP cells). The latest additions comprised the respiratory chain complex III and I inhibitors antimycin A (0.5 μM) and rotenone (0.5 μM). Finally, oxygen consumption was measured in different stressor-promoted respiration states, such as basal oxygen consumption, leak state, maximal respiratory capacity, and non-mitochondrial respiration. Units were expressed as oxygen flux per cell (pmol/s/cell). Results were reported as the mean ± SEM of three individual experiments.

### 3.14. Statistical Analysis

All experiments were performed in triplicate, and data are expressed as mean ± standard error of the mean (SEM). One-way analysis of variance (ANOVA) was used to compare treatment and control groups, followed by Bonferroni-adjusted multiple comparison tests. Nonparametric tests, such as the Kruskal–Wallis test, were used in cases where normality and homoscedasticity assumptions were not met. Nonparametric multiple comparisons were performed using the Mann–Whitney test. Using these considerations, a baseline analysis of the RWPE-1, LNCaP, and PC-3 cell lines prior to treatment with DCT and SFN was performed to establish a reference framework for identifying metabolic changes induced by these treatments.

In addition, dose-response curves and non-linear regression analyses were used to evaluate interactions between SFN and DCT using GraphPad Prism software (version 9.0, San Diego, CA, USA).

For the analysis of the effect of SFN and DCT treatments on apoptosis in the LNCaP and PC-3 cell lines, we chose the beta regression model. This model was selected because the variable of interest, the percentage of cell death, is bounded between 0 and 1, making it ideal for this type of analysis. The beta regression model effectively handles the asymmetric distribution of the data and provides accurate estimates of treatment effects at different stages of apoptosis (early and late). We calculated beta coefficients to quantify treatment efficacy, with significance at *p* < 0.05. These analyses were performed using the ‘betareg’ package in R software version 4.2.3 for Windows.

## 4. Conclusions

This study suggests that the combined treatment of SFN and DCT significantly enhances therapeutic effects in prostate cancer cell lines by modulating metabolic profiles and promoting apoptosis, especially in LNCaP cells. The SFN:DCT combination, administered at reduced doses, not only preserves efficacy but also minimizes toxicity, suggesting a potentially safer approach for patients. In LNCaP cells, this combined treatment induced a metabolic shift from glycolysis to OXPHOS and triggered apoptosis predominantly through the intrinsic pathway, as evidenced by caspase 3 activation. This dual impact—metabolic reprogramming and apoptosis—highlights the interaction between SFN and DCT; offering a promising avenue for treating early-stage prostate cancer.

In contrast, the more chemoresistant and aggressive PC-3 cells exhibited a comparatively muted apoptotic response, highlighting the challenges of targeting androgen-independent prostate cancer. The results suggest that while the SFN:DCT combination is effective in LNCaP cells, further research is needed to optimize strategies to overcome resistance in PC-3 cells. Overall, this study supports the SFN:DCT combination as a potential therapeutic strategy that influences metabolic modulation and apoptosis induction, opening up the possibility of improved prostate cancer treatments with reduced side effects.

## Figures and Tables

**Table 1:** Basal Metabolic and Oxidative Profiles in the RWPE-1, LNCaP, and PC-3 Cell Lines.

Parameters	RWPE-1 Mean ± SD	LNCaP Mean ± SD	PC-3 Mean ± SD	*p*-Value		
				RWPE-1 vs. LNCaP	RWPE-1 vs. PC-3	LNCaP vs. PC-3 Cells
Glucose (pg remaining/µL/cell)	2.98 ± 2.49	4.44 ± 3.78	0.41 ± 0.39	0.39	**0.04**	**0.01**
Lactate (nmol/L/cell)	0.64 ± 0.95	1.39 ± 0.19	0.08 ± 0.01	**0.00**	**0.00**	**0.00**
GSH (umoles/µL)	0.16 ± 0.002	0.22 ± 0.045	0.37 ± 0.047	0.13	0.13	**0.03**
GSSG (umoles/µL)	0.001879 ± 0.00013	0.0026 ± 0.0013	0.0032 ± 0.0008	0.80	0.13	0.72
GSH/GSSG	84.88 ± 4.63	139.33 ± 50.07	91.73 ± 16.70	0.33	1.00	0.33
ROS (RFI)	431,200.00 ± 53,204.51	12,417.5 ± 881.76	14,687.00 ± 1089.02	0.20	0.10	0.20
Mitochondrial mass (RFI)	1,040,000.00 ± 38,400.00	14,400.0 ± 11,900.00	160,000 ± 26,519.19	0.10	0.10	0.40
*SOD2* (Relative mRNA expression)	0.0000009 ± 0.0000004	0.0000003 ± 0.0000001	0.000005 ± 0.000002	0.10	0.10	0.20
*LDHA* (Relative mRNA expression)	0.000006 ± 0.000003	0.000013 ± 0.000006	0.000001 ± 0.0000005	0.08	0.10	0.08
Basal respiration (% OCR)	12.20 ± 3.04	8.67 ± 2.52	29.30 ± 5.03	0.40	0.20	0.10
Proton leak (% OCR)	10.0 ± 2.83	21.3 ± 5.86	27.00 ± 8.19	0.20	0.20	0.40
Max respiratory capacity (% OCR)	5.35 ± 1.90	8.67 ± 5.51	16.0 ± 5.29	0.80	0.20	0.20
Non mitochondrial respiration (% OCR)	6.00 ± 0.00	6.33 ± 2.52	15.33 ± 3.51	1.00	0.14	0.10

Mann–Whitney test *p* < 0.005 (5%). Bold for statistical significance difference. RFI: relative fluorescence intensity.

**Table 2:** Metabolic parameters and changes in the redox state of LNCaP and PC-3 cell lines after single and combined treatment with DCT and SFN.

Parameters	LNCaP Cells				PC-3 Cells			
	Treatments				Treatments			
	Control	SFN	DCT	SFN:DCT	Control	SFN	DCT	SFN:CT
	Mean ± SD	Mean ± SD	Mean ± SD	Mean ± SD	Mean ± SD	Mean ± SD	Mean ± SD	Mean ± SD
Glucose (pg remaining/µL/cell)	0.0035 ± 0.0003	0.00979 ± 0.0002	0.1045 ± 0.0034	0.0879 ± 0.0061	0.0005 ± 0.0001	0.0752 ± 0.0041	0.0690 ± 0.0002	0.0684 ± 0.055
Lactate (nmol/L/cell)	1.5695 ± 0.0021	1.0026 ± 0.0290	1.7761 ± 0.0432	1.2973 ± 0.0856	0.0955 ± 0.0039	1.4391 ± 0.1467	1.0941 ± 0.0669	1.4535 ± 1.1167
GSH (umoles/µL)	0.1848 ± 0.0331	0.0171 ± 0.0022	0.1084 ± 0.00259	1.2822 ± 0.0017	0.3685 ± 0.0252	0.9673 ± 0.0673	0.3339 ± 0.0082	0.5952 ± 0.0918
GSSG (umoles/µL)	0.002926 ± 0.001574	0.002255 ± 0.000133	0.002818 ± 0.000930	0.003946 ± 0.000664	0.0026 ± 0.0007	0.0168 ± 0.0024	0.0044 ± 0.0005	0.0039 ± 0.0017
Ratio GSH/GSSG	70.2873 ± 26.5011	7.5893 ± 0.5406	39.0867 ± 3.7018	72.5876 ± 12.6478	145.93 ± 46.43	57.74 ± 4.10	76.29 ± 11.04	172.47 ± 98.75
ROS (RFI*)	1.0 ± 0.2664	0.7873 ± 0.0498	1.4793 ± 0.1658	1.4391 ± 0.0988	1.0 ± 0.528	0.3716 ± 0.0065	0.7456 ± 0.0711	0.4600 ± 0.0192
Mitochondrial mass (RFI*)	1.0 ± 0.1311	**9.7365 ± 0.06823**	**5.4570 ± 1.4079**	**9.1872 ± 2.8282**	1.0 ± 0.1637	**0.4925 ± 0.1724**	**0.6605 ± 0.1039**	0.8903 ± 0.1359
*SOD2* (Relative mRNA expression)	1.0 ± 0.1708	1.8895 ± 0.02642	0.0525 ± 0.0062	0.1551 ± 0.0407	1.0 ± 0.1175	19.7233 ± 3.0496	0.6506 ± 0.1284	5.3560 ± 1.3269
*LDHA* (Relative mRNA expression)	1.0 ± 0.1708	1.5596 ± 0.2064	0.1274 ± 0.1294	0.1953 ± 0.0478	1.0 ± 0.1708	0.5062 ± 0.3967	0.9243 ± 0.0657	0.4789 ± 0.0855
Basal respiration (% OCR)	100 ± 27.47	81.95 ± 54.81	198.96 ± 39.10	66.50 ± 19.52	100 ± 30.32	88.89 ± 31.28	73.15 ± 44.17	59.26 ± 9.80
Proton leak (% OCR)	100 ± 63.55	164.42 ± 161.09	307.69 ± 203.26	161.54 ± 66.62	100 ± 56.83	112.50 ± 7.22	125.00 ± 72.35	97.92 ± 9.55
Max respiratory capacity (% OCR)	100 ± 29.04	242.31 ± 124.81	265.38 ± 196.04	181.73 ± 76.75	100 ± 17.16	95.45 ± 7.87	75.00 ± 28.66	61.36 ± 10.23
Non-mitochondrial respiration (% OCR)	100 ± 39.74	257.89 ± 165.85	431.58 ± 63.81	201.32 ± 86.84	100 ± 22.90	115.73 ± 30.83	115.76 ± 50.90	89.13 ± 7.53

**Bold** for significant differences when compared to a respective control cell line. RFI*: relative fluorescence intensity normalized to control (untreated cell).

**Table 3:** Beta Regression analysis of DCT, SFN, SFN:DCT Combination Treatments on Early and Late Apoptosis States, Redox, and Glycolytic Parameters in LNCaP Cells by Annexin Assay.

Compounds	Cell Death			Early Apoptosis			Late Apoptosis			Dead		
	Beta	95% CI ^1^	*p*-Value	Beta	95% CI ^1^	*p*-Value	Beta	95% CI ^1^	*p*-Value	Beta	95% CI ^1^	*p*-Value
Control	—	—		—	—		—	—		—	—	
DCT	**3**	**1.4**–**4.5**	<**0.001**	**2.9**	**1.6**–**4.3**	<**0.001**	**2.1**	**1.1**–**3.2**	<**0.001**	−0.12	−1.2–0.99	0.8
SFN	**3.5**	**1.9**–**5.1**	<**0.001**	**3.3**	**1.9**–**4.6**	<**0.001**	**2.7**	**1.7**–**3.7**	<**0.001**	0.29	−0.76–1.3	0.6
SFN: DCT	**3.1**	**1.6**–**4.6**	<**0.001**	**2.9**	**1.5**–**4.2**	<**0.001**	**2.2**	**1.2**–**3.3**	<**0.001**	−0.25	−1.4–0.88	0.7
**Parameters**												
Glucose	**58**	**43**–**72**	<**0.001**	**50**	**38**–**62**	<**0.001**	**22**	**12**–**32**	<**0.001**	−3.9	−12–4.5	0.4
Lactato	−0.96	−4.2–2.3	0.6	−1.7	−4.7–1.4	0.3	−0.85	−32–2.8. 1.1	0.4	0.73	−0.60–2.1	0.3
GSH	−0.76	−9.6–8.1	0.9	−3.2	−12–5.4	0.5	−0.44	−5.9–5.0	0.9	0.28	−3.5–4.1	0.9
GSSG	188	−763–1.140	0.7	43	−877–964	>0.9	194	−374–761	0.5	155	−241–551	0.4
GSH_GSSG	−0.01	−0.04–0.03	0.7	−0.02	−0.05–0.02	0.3	−0.01	−0.03–0.02	0.6	0	−0.01–0.02	>0.9
ROS	0.56	−1.7–2.8	0.6	0.71	−1.4–2.8	0.5	0.57	−0.93–2.1	0.5	−0.34	−1.7–1.0	0.6
Mitochondrial mass	**0.28**	**0.14**–**0.42**	<**0.001**	**0.24**	**0.11**–**0.36**	<**0.001**	**0.15**	**0.07**–**0.24**	<**0.001**	−0.01	−0.10–0.08	0.9
*SOD2*	−0.09	−1.1–0.88	0.9	−0.2	−1.1–0.71	0.7	−0.11	−0.76–0.54	0.7	0.2	−0.38–0.77	0.5
*LDHA*	−0.3	−1.5–0.91	0.6	−0.47	−1.6–0.66	0.4	−0.44	−1.2–0.36	0.3	0.17	−0.56–0.90	0.6
Basal respiration	0	−0.01–0.01	0.7	0.01	−0.01–0.02	0.3	0	−0.01–0.01	0.7	0	−0.01–0.00	0.3
Proton leak	0	0.00–0.01	0.3	0	0.00–0.01	0.1	0	0.00–0.00	0.3	0	−0.01–0.00	0.12
Max respiratory capacity	0	0.00–0.01	0.4	0	0.00.–0.01	0.2	0	0.00–0.00	0.4	0	0.00–0.00	0.8
Non-mitochondrial respiration	**0.01**	**0.00**–**0.01**	**0.018**	**0.01**	**0.00**–**0.01**	**0.002**	0	0.00–0.01	0.058	0	0.00–0.00	0.5

^1^ CI = Confidence Interval. Bold for statistical significance difference, Mann–Whitney test *p* < 0.005 (5%).

**Table 4:** Beta Regression analysis of DCT, SFN, SFN:DCT Combination Treatments on Early and Late Apoptosis States, Redox, and Glycolytic Parameters in PC-3 Cells by Annexin Assay.

Compounds	Cell Death			Early Apoptosis			Late Apoptosis			Dead		
	Beta	95% CI ^1^	*p*-Value	Beta	95% CI ^1^	*p*-Value	Beta	95% CI ^1^	*p*-Value	Beta	95% CI ^1^	*p*-Value
Control	—	—		—	—		—	—		—	—	
DCT	**2.4**	**1.8**–**3.0**	<**0.001**	**2.1**	**0.99**–**3.1**	<**0.001**	0.79	−0.46–2.0	0.2	−0.68	−1.8–0.41	0.2
SFN	**2.0**	**1.4**–**2.6**	<**0.001**	**1.9**	**0.85**–**3.0**	<**0.001**	0.28	−1.0–1.6	0.7	−0.75	−1.8–0.35	0.2
SFN:DCT	**2.0**	**1.4**–**2.6**	<**0.001**	**1.8**	**0.77**–**2.9**	<**0.001**	0.50	−0.78–1.8	0.4	−0.66	−1.7–0.43	0.2
Parameters												
Glucose	**33**	**23**–**42**	<**0.001**	**30**	**12**–**47**	<**0.001**	5.1	−16–26	0.6	−11	−28–5.5	0.2
Lactato	**1.5**	**0.84**–**2.2**	<**0.001**	**1.6**	**0.66**–**2.5**	<**0.001**	0.13	−1.0–1.3	0.8	−0.68	−1.6–0.23	0.14
GSH	1.4	−0.67–3.5	0.2	1.8	−0.46–4.0	0.12	−0.04	−2.5–2.4	>0.9	−0.78	−3.1–1.5	0.5
GSSG	62	−28–152	0.2	67	−32–165	0.2	21	−86–127	0.7	−7.5	−107–92	0.9
GSH/GSSG	0.00	−0.01–0.01	0.5	0.00	−0.01–0.01	0.8	−0.01	−0.02–0.00	0.2	0.00	−0.01–0.01	0.5
ROS	**−2.2**	**−3.9**–**0.47**	**0.012**	**−2.1**	**−4.1**–**0.10**	**0.039**	−1.1	−3.2–0.91	0.3	0.51	−1.4–2.4	0.6
Mitochondrial mass	**−2.0**	**−3.3**–**0.68**	**0.003**	**−2.0**	**−3.6**–**0.49**	**0.010**	−0.42	−2.1–1.3	0.6	1.1	−0.37–2.7	0.14
*SOD2*	0.03	−0.03–0.09	0.3	0.03	−0.04–0.10	0.4	0.03	−0.03–0.09	0.3	0.01	−0.05–0.07	0.7
*LDHA*	−1.3	−2.8–0.23	0.10	−1.5	−3.2–0.18	0.079	0.10	−1.6–1.8	>0.9	0.63	−0.97–2.2	0.4
Basal respiration	−0.01	−0.02–0.00	0.2	−0.01	−0.03–0.00	0.13	0.00	−0.02–0.01	0.9	0.01	−0.01–0.02	0.2
Proton leak	0.00	0.00–0.01	0.3	0.00	−0.01–0.01	0.5	0.00	−0.01–0.01	>0.9	0.00	−0.01–0.01	0.7
Max respiratory capacity	−0.02	−0.04–0.00	0.068	−0.02	−0.04–0.00	0.11	−0.01	−0.03–0.01	0.5	0.01	−0.01–0.03	0.5
Non-mitochondrial respiration	0.00	−0.01–0.01	>0.9	0.00	−0.02–0.01	0.7	0.00	−0.01–0.02	0.6	0.00	−0.01–0.02	0.6

^1^ CI = Confidence Interval. **Bold** for statistical significance difference, Mann–Whitney test *p* < 0.005 (5%).

## Data Availability

The raw data supporting the conclusions of this article will be made available by the authors on request.

## Supplementary Materials

The following supporting information can be downloaded at: https://www.mdpi.com/article/10.3390/ijms26031013/s1.
