# Supplementary material for: Combination of Low-Dose Sulforaphane and Docetaxel on Mitochondrial Function and Metabolic Reprogramming in Prostate Cancer Cell Lines
Source: Int J Mol Sci. 2025 Jan 24;26(3):1013. doi: 10.3390/ijms26031013 (PMC11817897; doi:10.3390/ijms26031013)
Supplement: Supplementary file 1 [file ijms-26-01013-s001.zip › ijms-3331424-supplementary.pdf]

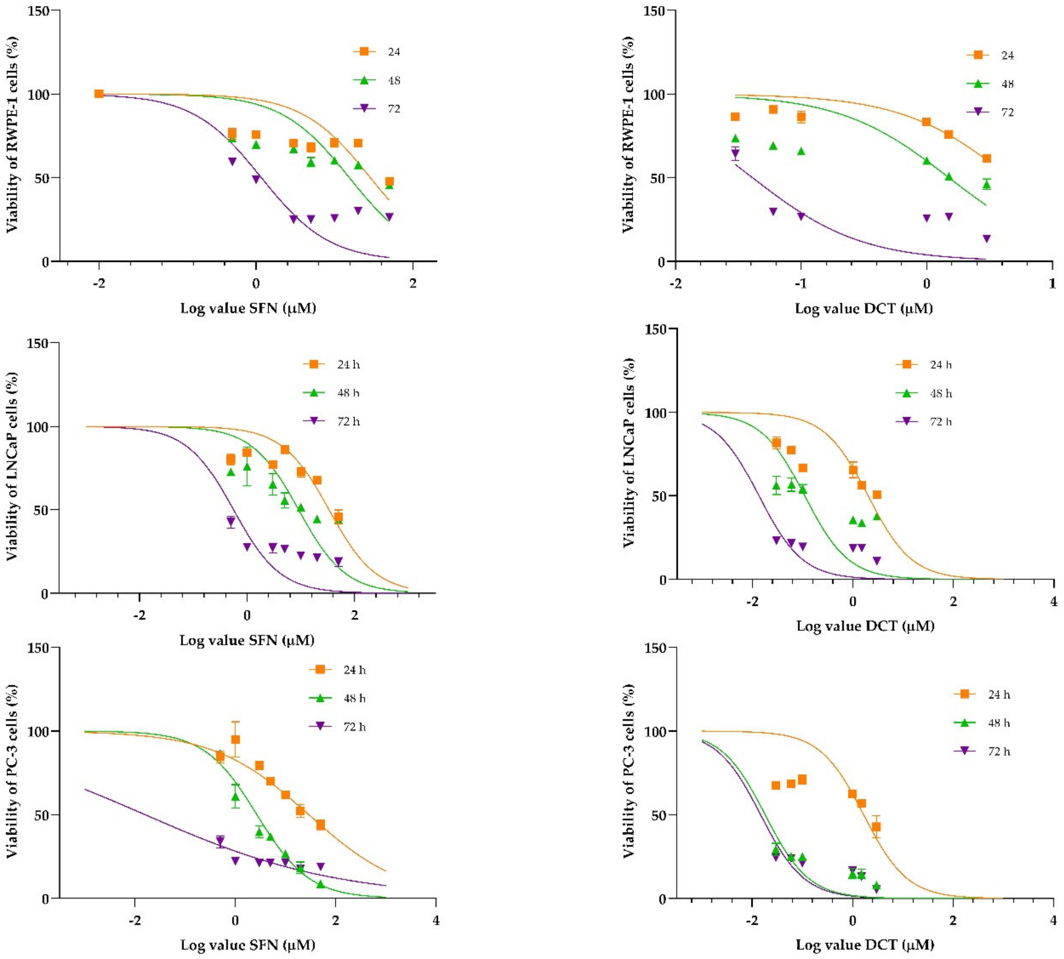

Supplementary Figure S1. Dose-response curves for the RWPE-1, LNCaP, and PC-3 lines, evaluated by CV staining, treated with SFN and DCT for 24, 48, and 72 hours. The data are shown as the mean ± SD from n = 3 independent experiments.

Supplementary Table S1. Cell viability profiles of RWPE-1, LNCaP and PC-3 lines, evaluated by CV staining, treated with SFN and DCT for 24, 48 and 72 hours.

Cell lines	IC50 for substances					
	**SFN (μM)**			**DCT (μM)**		
	**Time (h)**					
	**24 h**	**48 h**	**72 h**	**24 h**	**48 h**	**72h**
**RWPE-1**	169.2	40.55	0.661	26.06	2.303	0.02283
**LNCaP**	67.86	13.08	$4.443\text{x}10^{-4}$	3.686	0.1315	$8.766\text{x}10^{-14}$
**PC-3**	29.15	2.230	$7.865\text{x}10^{-8}$	36.37	0.01	$1.720\text{x}10^{-6}$

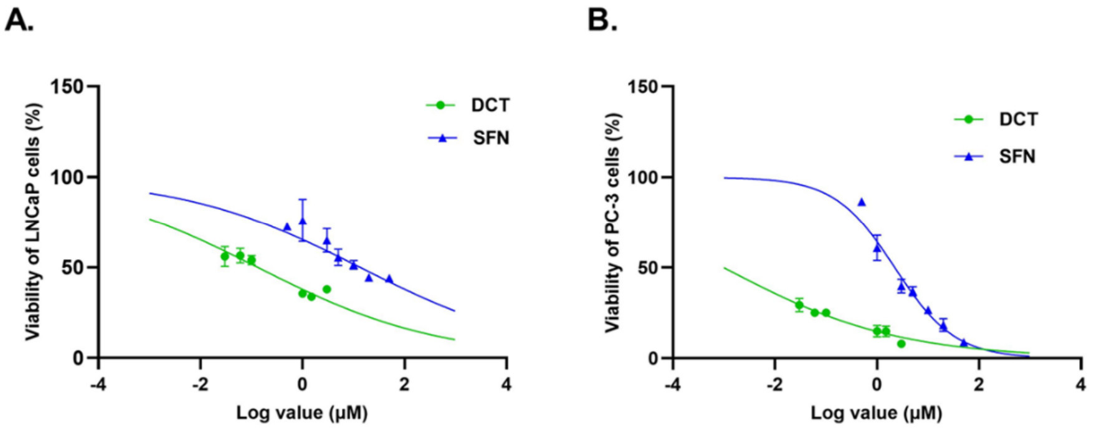

Supplementary Figure S2. Power relationships generated by SFN and DCT on LNCaP and PC-3 cells. (a) Constant power relationships between SFN and DCT, on LNCaP viability, equal slopes are observed for all data sets **** $p> 0.0001$. (b) Constant power relationships between SFN and DCT, on PC-3 viability, different slopes are observed for each data set ****$p < 0.0001$. The data are shown as the mean ± SD from n = 3 independent experiments.

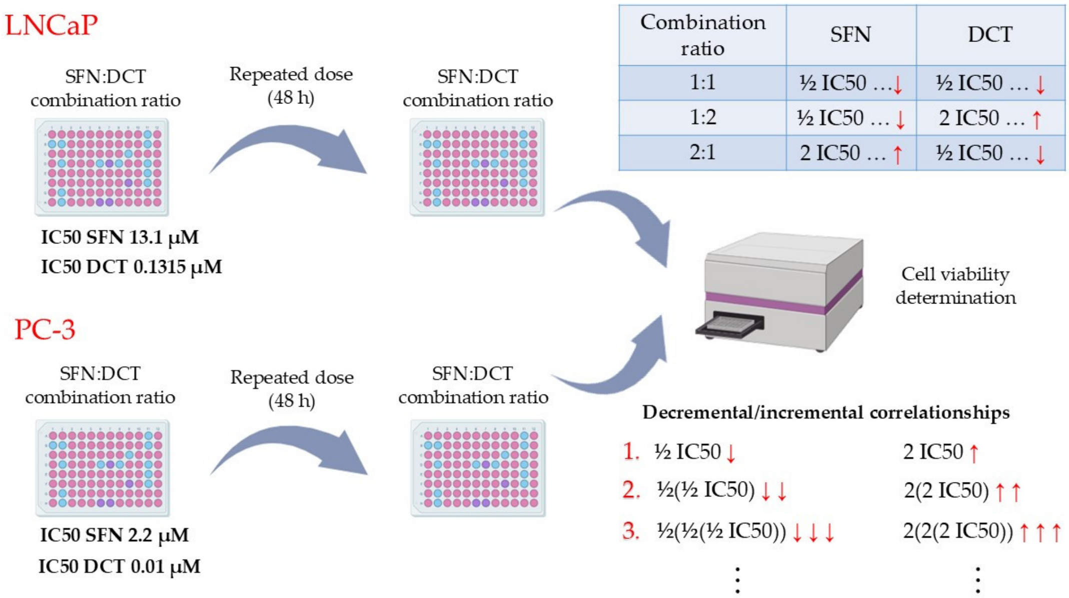

Supplementary Figure S3. Experimental design for SFN:DCT combination ratios in LNCaP and PC-3 cells. Assays were conducted using a 1:1 ratio based on the IC50 values obtained, specifically 1/2 IC50 SFN: 1/2 IC50 DCT. For LNCaP cells, the combination included 6.55 μM SFN and 0.0675 μM DCT, while for PC-3 cells, 1.1 μM SFN and 0.0005 μM DCT were used. All treatments were performed with an incubation period of 48 hours to evaluate the effects on both prostate cancer cell lines. All combination ratios, including the 1:1 ratio, are based on IC50 values and demonstrate incremental and decremental relationships between the concentrations of SFN and DCT.

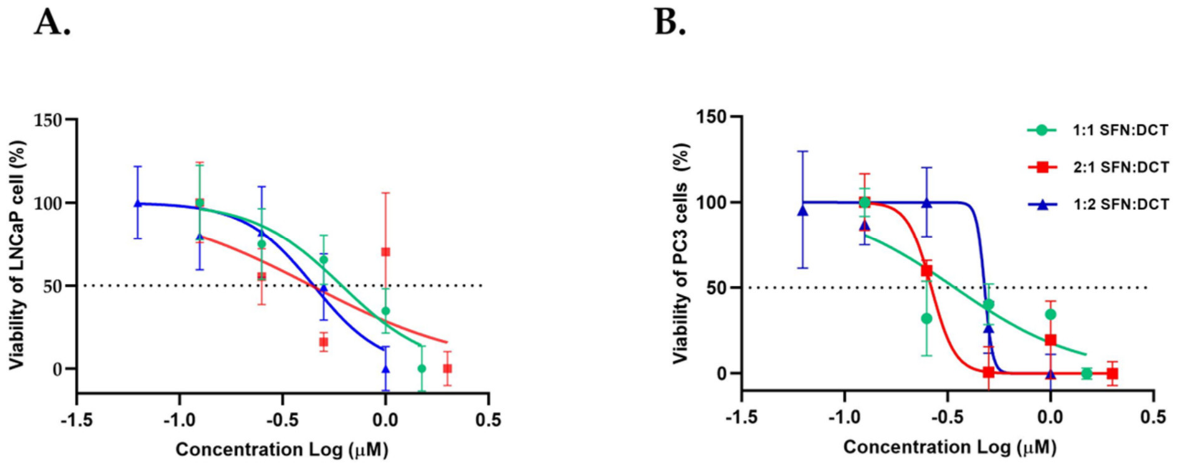

Supplementary Figure S4. The effects of SFN and DCT interaction administered at fixed concentrations of SFN: DCT 1:1, 2:1, and 1:2 in prostate tumor lines by CV assay. Results are expressed as dose-response curves. The data are shown as the mean ± SD from n = 3 independent experiments.

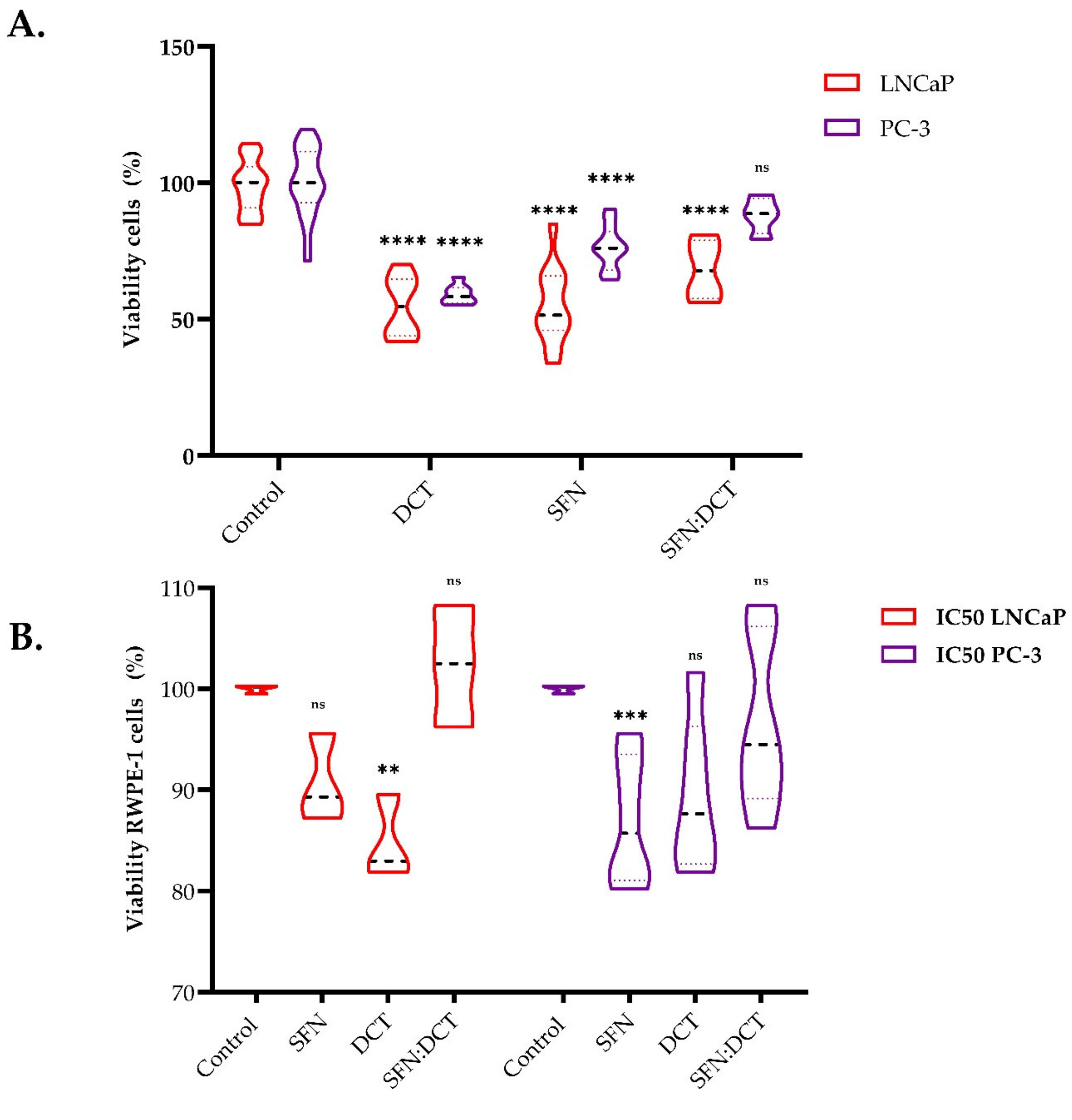

Supplementary Figure S4. A. The impact of SFN, DCT, and the SFN:DCT combination (at half IC50) on prostate cancer cell lines LNCaP and PC-3. **B.** The influence of SFN, DCT, and the SFN-DCT combination (at half IC50) on prostate cancer cell lines LNCaP and PC-3. The SFN:DCT combination (at half IC50) was also tested on non-tumorigenic prostate cells, RWPE-1. The results indicated a statistically significant effect at ****$p < 0.001$* ****$p < 0.0001$*, while no significant effect was observed at ns: not significant. The data are shown as the mean ± SD from n = 3 independent experiments.
